# Causal Associations of Obesity With the Intervertebral Degeneration, Low Back Pain, and Sciatica: A Two-Sample Mendelian Randomization Study

**DOI:** 10.3389/fendo.2021.740200

**Published:** 2021-12-08

**Authors:** Jingzhu Zhou, Jiarui Mi, Yu Peng, Huirong Han, Zhengye Liu

**Affiliations:** ^1^ School of Anesthesiology, Weifang Medical University, Weifang, China; ^2^ Master Programme in Biomedicine, Karolinska Institutet, Stockholm, Sweden; ^3^ Department of Orthopedics, Zhongnan Hospital of Wuhan University, Wuhan, China

**Keywords:** Mendelian randomization, obesity, intervertebral disc degeneration, sciatica, lower back pain

## Abstract

The role of obesity in the development of dorsopathies is still unclear. In this study, we assessed the associations between body mass index (BMI) and several dorsopathies including intervertebral disc degeneration (IVDD), low back pain (LBP), and sciatica by using the Mendelian randomization method. We also assessed the effect of several obesity-related traits on the same outcomes. Single-nucleotide polymorphisms associated with the exposures are extracted from summary-level datasets of previously published genome-wide association studies. Summary-level results of IVDD, LBP, and sciatica were from FinnGen. In our univariable Mendelian randomization analysis, BMI is significantly associated with increased risks of all dorsopathies including sciatica (OR = 1.33, 95% CI, 1.21–1.47, p = 5.19 × 10^-9^), LBP (OR = 1.28, 95% CI, 1.18–1.39, p = 6.60 × 10^-9^), and IVDD (OR = 1.23, 95% CI, 1.14–1.32, p = 2.48 × 10^-8^). Waist circumference, hip circumference, whole-body fat mass, fat-free mass, and fat percentage, but not waist–hip ratio, were causally associated with increased risks of IVDD and sciatica. Higher hip circumference, whole-body fat mass, fat-free mass, and fat percentage increased the risk of LBP. However, only whole-body fat-free mass remained to have a significant association with the risk of IVDD after adjusting for BMI with an odds ratio of 1.57 (95% CI, 1.32–1.86, p = 2.47 × 10^-7^). Proportions of BMI’s effect on IVDD, sciatica, and LBP mediated by leisure sedentary behavior were 41.4% (95% CI, 21.8%, 64.8%), 33.8% (95% CI, 17.5%, 53.4%), and 49.7% (95% CI, 29.4%, 73.5%), respectively. This study provides evidence that high BMI has causal associations with risks of various dorsopathies. Weight control is a good measure to prevent the development of dorsopathies, especially in the obese population.

## Introduction

As one of the most common chronic disorders, obesity has raised alarming interest worldwide due to its significant impact on social cost and life expectancy (1). Obesity is associated with multiple musculoskeletal disorders including osteoarthritis, intervertebral disc disease (IVDD), sciatica, and low back pain (LBP) and thus reduced the motility and life quality of the patients (1–3).

IVDD is a common degenerative disorder characterized by the progressive loss of proteoglycans and water content in the nucleus pulposus (NP) and may eventually result in the breakdown of the discs in between the vertebras (4, 5). Degenerated discs are more prone to be out-pouching (herniation) and may press against spinal nerves and the nerve root. Although asymptomatic in various cases, disc degeneration is known to be associated with disc herniation or prolapse, low back pain, and sciatica (6–8). When nerves are irritated in the lower back due to IVDD, this condition is referred to as lumbar radiculopathy. When this happens to the L4-S1 nerve roots, it leads to a commonly recognized pain called sciatica (8, 9).

The etiology and pathophysiology of IVDD are still not fully characterized yet. It may be the result of a combination of genetic background and environmental factors including aging, overloading, physical activity, and smoking (10–12). Endplate defect has been shown to be the major initiating factor contributing to IVDD and Modic changes (13–17). Previous studies also noticed an association between obesity and increased IVDD risk (18–20). Rade et al. suggested that high BMI may increase the risk of IVDD through predisposing the endplate defect (16). However, these studies are mostly cohort or cross-sectional studies and are unable to conclude a causal relationship. Furthermore, there are studies that provided conflicting evidence against the association (21).

Mendelian randomization (MR) is a method that uses single-nucleotide polymorphism (SNP) as instrumental variables for estimating the causal effect of an exposure on an outcome (22, 23). Since genetic variants are randomly assigned, the study is protected from potential confounding factors (24). Besides, since the SNP alleles are assigned during the meiosis before the onset of the disease, the risk of reverse causality is minimized. The aim of the study is to assess the causal associations between body mass index (BMI)/obesity-related traits and dorsopathies including IVDD, LBP, and sciatica with a two-sample MR approach.

## Material and Methods

### Study Design

The diagram of the study design with the three assumptions of Mendelian randomization study is displayed in **Supplementary Figure 1** (24). First, the genetic variants should have a direct effect on the risk of the outcomes. Second, the genetic variants are not associated with any confounding factors. Lastly, the effects of the genetic variants on the health outcomes are only mediated by the exposures. In the current study, we assessed the causal effects of BMI and body composition on several dorsopathies by using publicly available datasets from large genome-wide association studies (GWAS). Summary-level data of genetic instruments associated with the exposures including BMI and obesity-related traits were extracted from publicly available GWAS (25–27). Summary-level GWAS results of the outcomes (IVDD, sciatica with lumbago, LBP) were obtained from FinnGen consortium R4 enrolling 176,899 subjects (https://www.finngen.fi/en). For the MR analysis, firstly, we conducted univariable MR to investigate the causal associations of obesity-related factors on the risk of several dorsopathies. Next, since people with dorsopathies are prone to have less physical activity and gain weight, we performed bidirectional MR analysis to investigate whether BMI and dorsopathies follow a bidirectional manner. Lastly, we did multivariable MR analysis with each anthropometric trait adjusted with BMI. We also performed a mediation analysis assessing the proportion of BMI’s effect on the outcomes mediated by potential confounders (leisure sedentary behavior).

### Data Sources

Genetic instrument variables for the exposures including BMI (sample size N = 681,275), waist circumference (N = 232,101), hip circumference (N = 213,038), waist–hip ratio (212,244), whole-body fat mass (N = 454,137), whole-body fat-free mass (N = 454,850), and whole-body fat percentage (331,117) were selected at a genome-wide significant level (p < 5 × 10^–8^) from the GIANT consortium or UK Biobank GWASs in the European population. The PLINK clumping method was used for calculating the linkage disequilibrium (LD) among the selected SNPs. The criteria for LD were defined as SNPs with R^2^ > 0.01 and physical distance within 5,000 kb. The SNPs identified as in LD were excluded from the subsequent analyses. To test for weak instruments, the mean F-statistics of the exposures were generated with a previously described approximation method (28). F-statistics for all included exposures are above 10 (**Supplementary Table 1**). Detailed information of the included summary results for the exposures are shown in **Supplementary Table 1**.

Dorsopathies discussed in this study include IVDD (15,565 cases and 134,889 controls), sciatica (6,827 cases and 134,889 controls), and LBP (9,917 cases and 134,889 controls). The summary results of all the outcomes were obtained from the FinnGen consortium. Detailed information of the outcomes including definitions and inclusion/exclusion criteria is presented in **Supplementary Table 2**. Heritability of the included exposures and outcomes is also presented in **Supplementary Tables 1**, **2**. In order to study the effect of bidirectional causality, we also performed MR analyses to assess the effect of IVDD, sciatica, and LBP on BMI.

### Statistical Analysis

Briefly, univariable MR analysis was first used to assess the causal associations of the selected exposures with outcomes respectively. In detail, the inverse-variance–weighted (IVW) method was used as the main analysis for MR. We used the random-effect IVW method when significant heterogeneity was noticed (p < 0.1 in the heterogeneity test, **Supplementary Table 3**); otherwise, a fixed-effect IVW was used. The weighted median method was also used since it can provide consistent estimates when up to 50% of the weight in the analysis were originated from invalid instrumental variables (28). In addition, in case the instrumental variable assumptions cannot be fully satisfied, we also performed the MR-Egger method and MR-PRESSO (Mendelian Randomization Pleiotropy RESidual Sum and Outlier) method to detect potential pleiotropy and outliers (29, 30). The MR-Egger method can identify and correct potential pleiotropy (p for intercept < 0.05) and gives a consistent estimate (29). The outlier test in the MR-PRESSO can detect possible outliers and provide adjusted results after excluding the outliers and thus correcting for the horizontal pleiotropy. Next, to assess whether the causal effects of other obesity-related traits on the outcomes were mediated by BMI, we performed multivariable MR analysis to adjust for the effects of BMI with multivariable random-effect IVW analysis (30). We further performed Generalized Summary-data-based Mendelian Randomization (GSMR) to assess the effects of BMI on the outcomes, by using samples from “1000 Genome Project Phase 1” as reference sample for LD estimation (31, 32). Lastly, we calculated the proportion of BMI’s effect on the outcomes mediated by potential confounders by using a mediation analysis (33). Funnel plots and scatter plots of the MR analyses were used to visually assess horizontal pleiotropy and heterogeneity (**Supplementary Figures 2**–**43**). All statistical analyses were two-sided. A p-value was considered statistically significant when less than 0.002 (0.05/21 adjusted with the Bonferroni method) and was considered suggestively significant between 0.002 and 0.05. All analyses were conducted with R (version 4.0.2), TwoSampleMR (0.5.5), Mendelian Randomization (0.5.0), and MR-PRESSO package (30, 34–36).

## Results

### Univariable Mendelian Randomization

**Figure 1:**
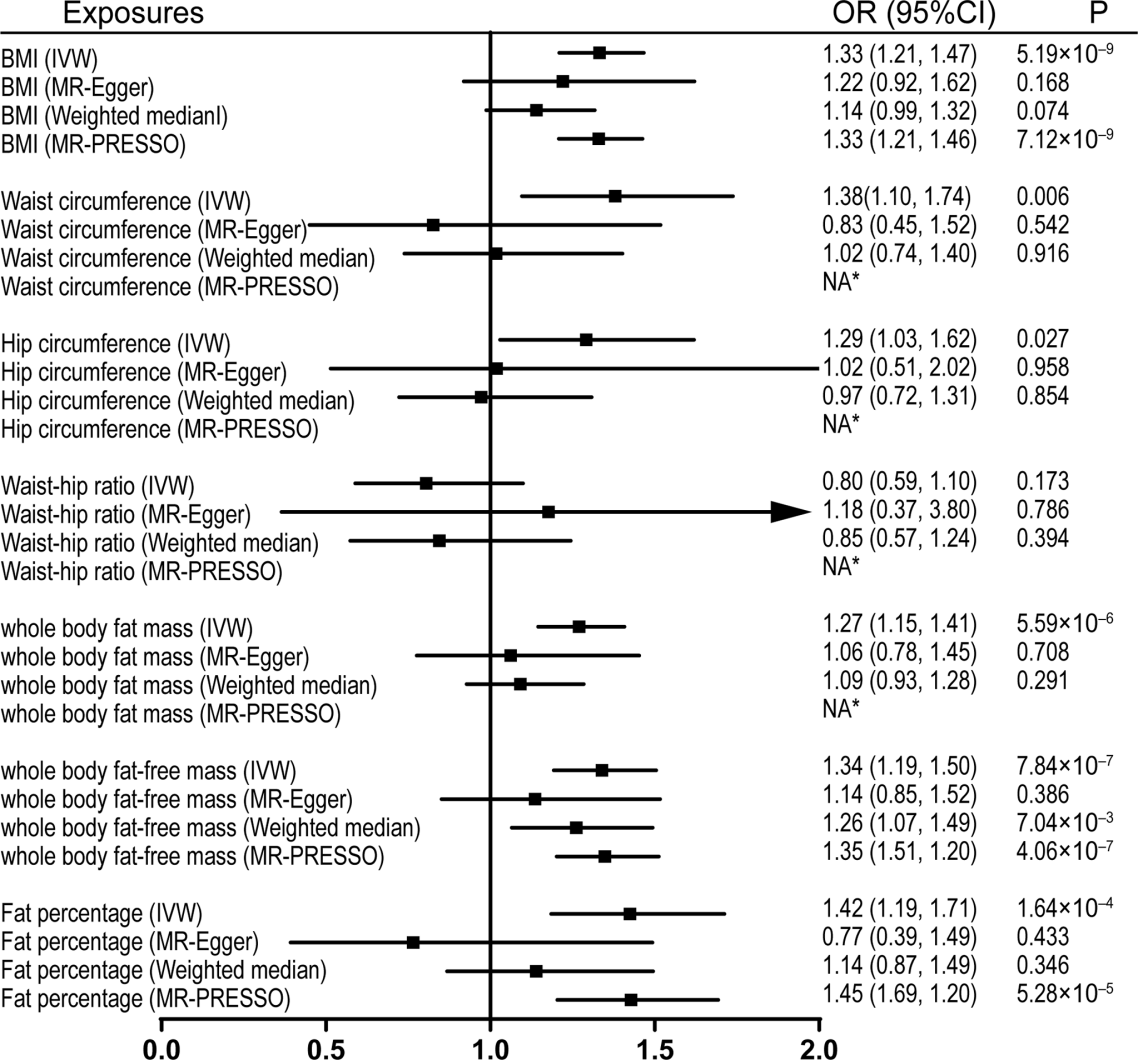
Effects of obesity-related exposures on sciatica. Results from univariable MR analyses showing the effects of genetically proxied BMI and obesity-related traits on sciatica with IVW, MR-Egger, weighted median, and MR-PRESSO methods. *No outlier detected. IVW, inverse-variance weighted; BMI, body mass index; OR, odds ratio; CI, confidence interval; MR, Mendelian randomization. NA, not available.

**Figure 2:**
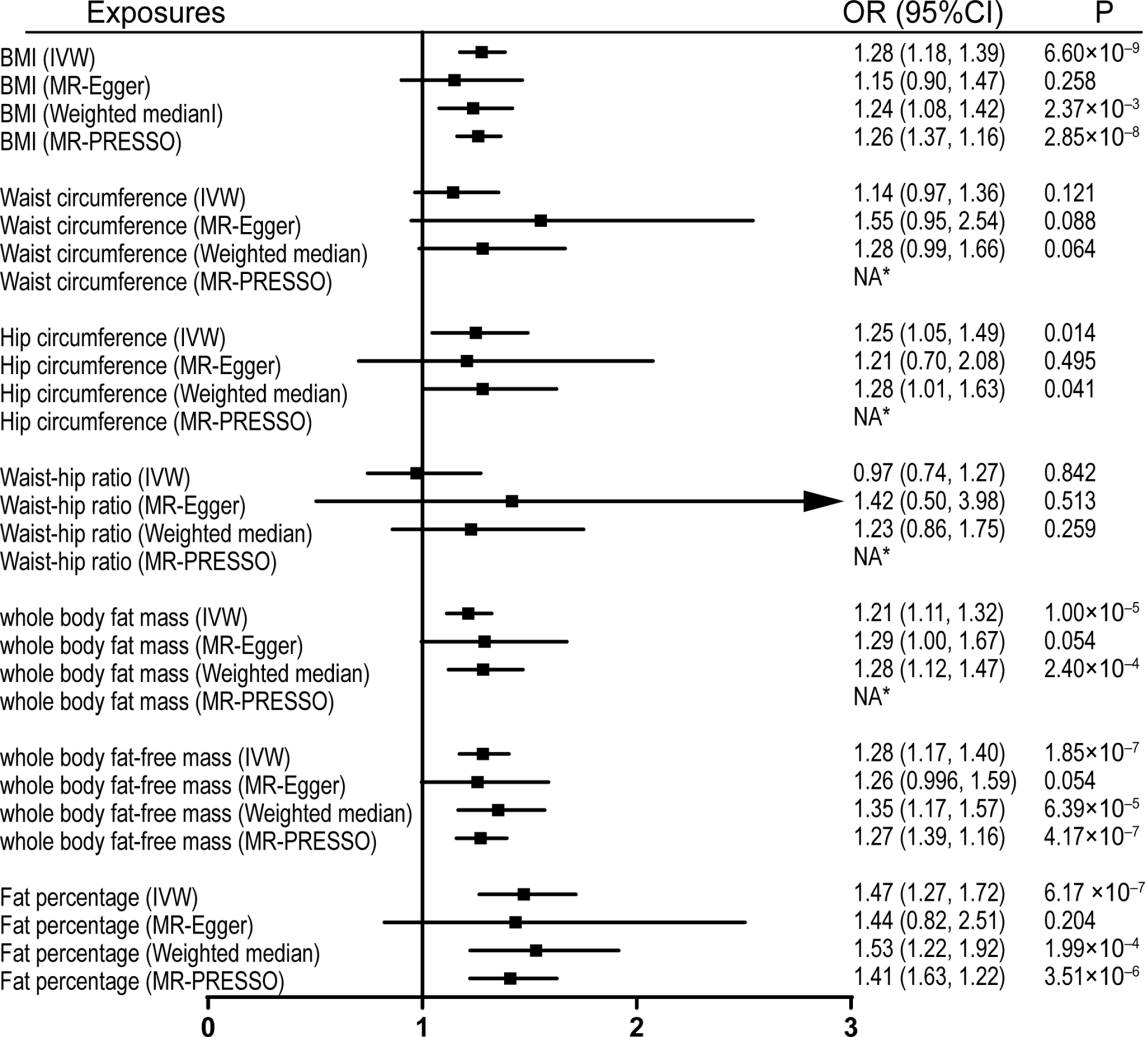
Effects of obesity-related exposures on LBP. Results from univariable MR analyses showing the effects of genetically proxied BMI and obesity-related traits on LBP with IVW, MR-Egger, weighted median, and MR-PRESSO methods. *No outlier detected. IVW, inverse-variance weighted; BMI, body mass index; LBP, low back pain; OR, odds ratio; CI, confidence interval; MR, Mendelian randomization. NA, not available.

**Figure 3:**
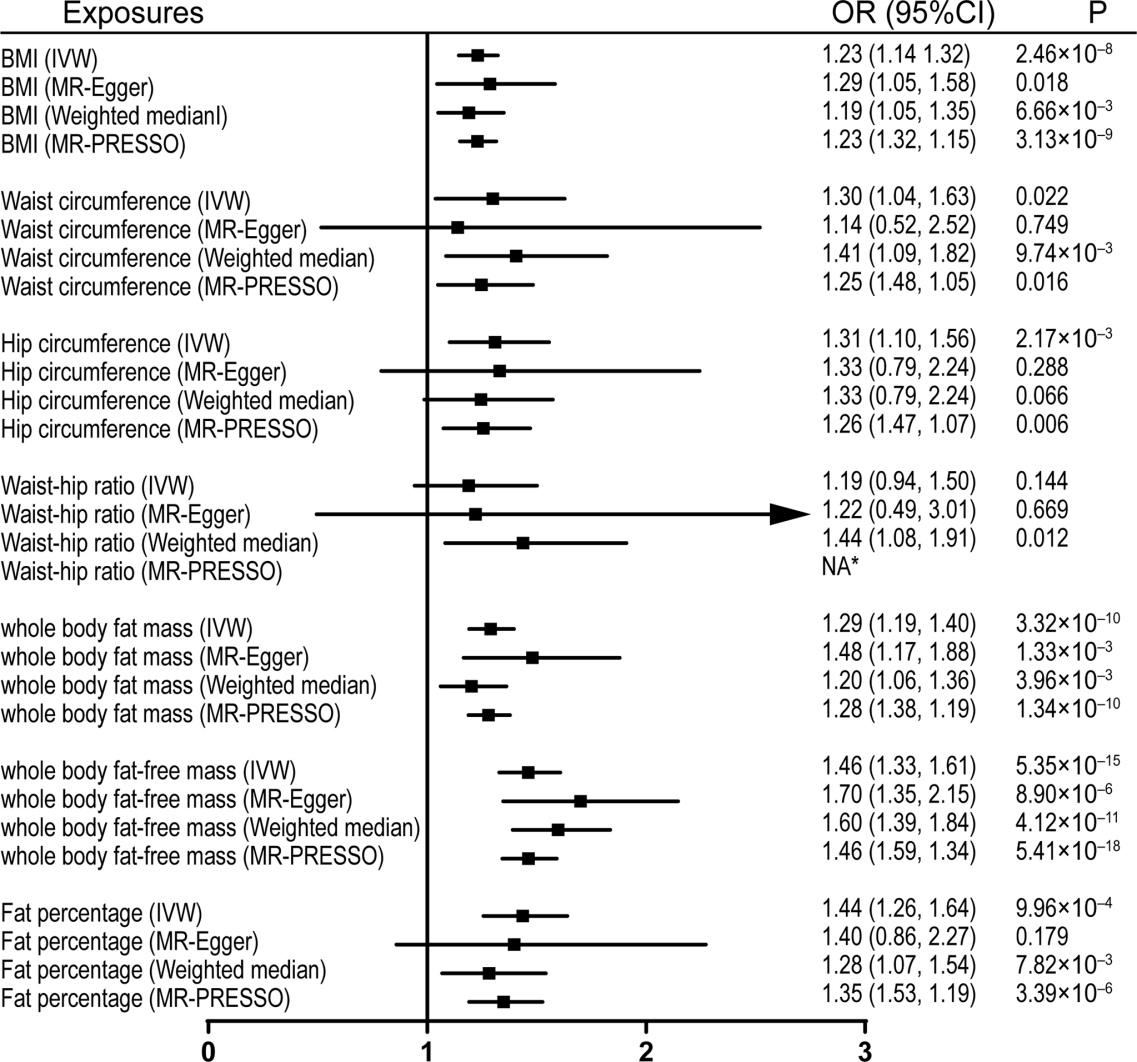
Effects of obesity-related exposures on IVDD. Results from univariable MR analyses showing the effects of genetically proxied BMI and obesity-related traits on IVDD with IVW, MR-Egger, weighted median, and MR-PRESSO methods. *No outlier detected. IVW, inverse-variance weighted; BMI, body mass index; IVDD, intervertebral disc degeneration; OR, odds ratio; CI, confidence interval; MR, Mendelian randomization. NA, not available.

Univariable MR was firstly performed for assessing the effects of levels of BMI, and other obesity-related traits on IVDD, sciatica, and LBP (**Supplementary Table 3**). Of note, genetically proxied BMI is significantly associated with all the outcomes. For the 1-SD increase in BMI level, the odds ratios were 1.33 (95% CI, 1.21–1.47, p = 5.19 × 10^-9^) for sciatica and 1.28 (95% CI, 1.18–1.39, p = 6.60 × 10^-9^) for LBP with the IVW method and 1.23 (95% confidence interval [CI], 1.14–1.32, p = 2.48 × 10^-8^) for IVDD (**Figures 1**–**3**). After excluding outliers with MR-PRESSO, the causal association of BMI with the outcomes remained significant. Among other obesity-related traits, waist circumference, hip circumference, whole-body fat mass, fat-free mass, and fat percentage, but not waist-hip ratio, are causally associated with an increased risk of IVDD with random-effect IVW and remained consistent after MR-PRESSO correction for outliers (**Figure 1**). Similar associations were observed for sciatica; all obesity-related traits except the waist–hip ratio increased the risk of sciatica with both the IVW method and MR-PRESSO adjustment (**Figure 2**). Hip circumference is also positively correlated with the risk of LBP; by contrast, waist circumference and waist–hip ratio are not (**Figure 3**). With genetically predicted body fat measures, we showed causal associations of body fat mass, fat-free mass, and fat percentage with the risk of LBP (**Figure 3**). No significant pleiotropy was detected in all the analyses (**Supplementary Table 3**).

### Bidirectional Mendelian Randomization

Two SNPs were identified as significantly associated with IVDD and available as instrumental variables (rs1431196 [p = 4.69 × 10^-8^], rs4148928 [p = 4.28 × 10^-9^]). IVDD was positively correlated with BMI level (beta = 0.057, 95% CI, 0.024–0.089, p = 6.00×10^-4^) by using a fixed-effect IVW method. We searched for the two SNPs in PhenoScanner (http://www.phenoscanner.medschl.cam.ac.uk/), and no horizontal pleiotropy was noticed. Only one SNP was significantly associated with sciatica (rs568306051 [p = 3.80 × 10^-8^]), but not available as a genetic instrument for MR analysis, and no SNP was found significantly associated with LBP.

### Multivariable Mendelian Randomization and Mediation Analysis

**Figure 4:**
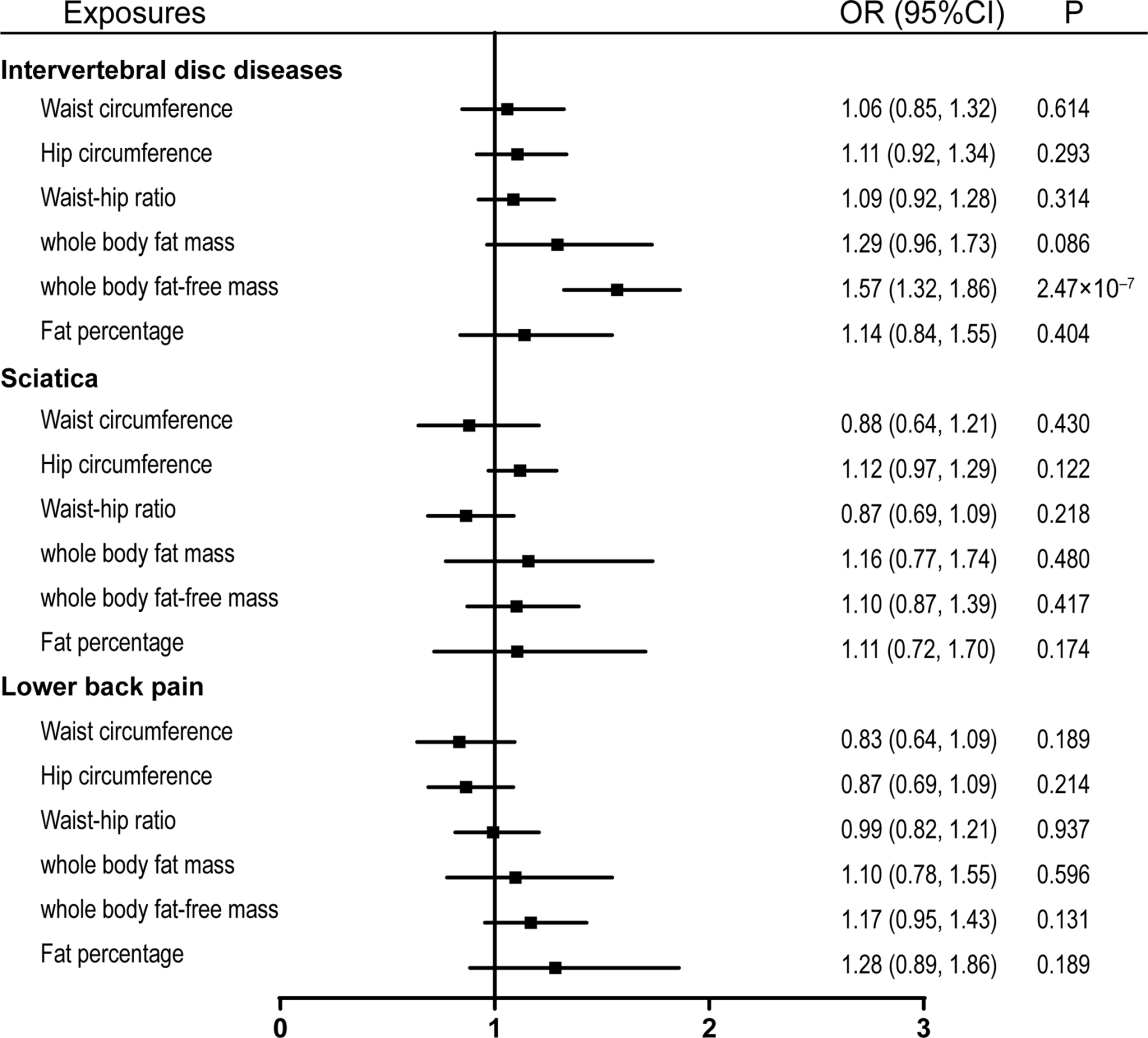
Effects of obesity-related exposures adjusted for BMI. Results from multivariable MR analyses showing the effects of obesity-related traits on IVDD, sciatica, and LBP after adjusting for the effect of BMI. BMI, body mass index; OR, odds ratio; CI, confidence interval; MR, Mendelian randomization; IVDD, intervertebral disc degeneration; LBP, low back pain.

Due to the high relevance of the included obesity-related traits with BMI, we further used multivariable MR analysis to adjust the effects of these traits for BMI. Only whole-body fat-free mass remained significantly associated with the risk of IVDD after adjusting for BMI with an odds ratio of 1.57 (95% CI, 1.32–1.86, p = 2.47 × 10-7) (**Figure 4**).

We further calculated the proportion of BMI’s effect on the outcomes mediated by leisure sedentary behavior (proxied with television watching) by using mediation analysis. The proportions of BMI’s effect on IVDD, sciatica, and LBP mediated by sedentary behavior were 41.4% (95% CI, 21.8%, 64.8%), 33.8% (95% CI, 17.5%, 53.4%), and 49.7% (95% CI, 29.4%, 73.5%) respectively (**Table 1**).

**Table 1:** Proportion of BMI’s effect on IVDD, sciatica, and LBP mediated by sedentary behavior.

Exposure	Mediator	outcomes	Total effect*^a^*	Effect X*^b^*	Effect M*^c^*	Mediation effect*^d^*	Mediated proportion
			Effect size (95% CI)	Effect size (95% CI)	Effect size (95% CI)	Effect size (95% CI)	(%)(95% CI)
BMI	Sedentary	IVDD	0.21 (0.14, 0.27)	0.20 (0.13, 0.27)	0.42 (0.26, 0.59)	0.09 (0.05, 0.13)	41.4% (21.8%, 64.8%)
BMI	Sedentary	Sciatica	0.28 (0.19, 0.38)	0.20 (0.13, 0.27)	0.47 (0.28, 0.67)	0.10 (0.05, 0.15)	33.8% (17.5%, 53.4%)
BMI	Sedentary	LBP	0.23 (0.15, 0.31)	0.20 (0.13, 0.27)	0.56 (0.40, 0.73)	0.11 (0.07, 0.17)	49.7% (29.4%, 73.5%)

BMI, body mass index; IVDD, intervertebral disc degeneration; LBP, low back pain.

^a^Total effect: the effect of exposures on outcomes.

^b^Effect X: the effect of exposures on mediators.

^c^Effect M: the effect of mediators on outcomes.

^d^Mediation effect: the effect of exposures on outcomes via mediators.

### Generalized Summary-Data-Based Mendelian Randomization

**Figure 5:**
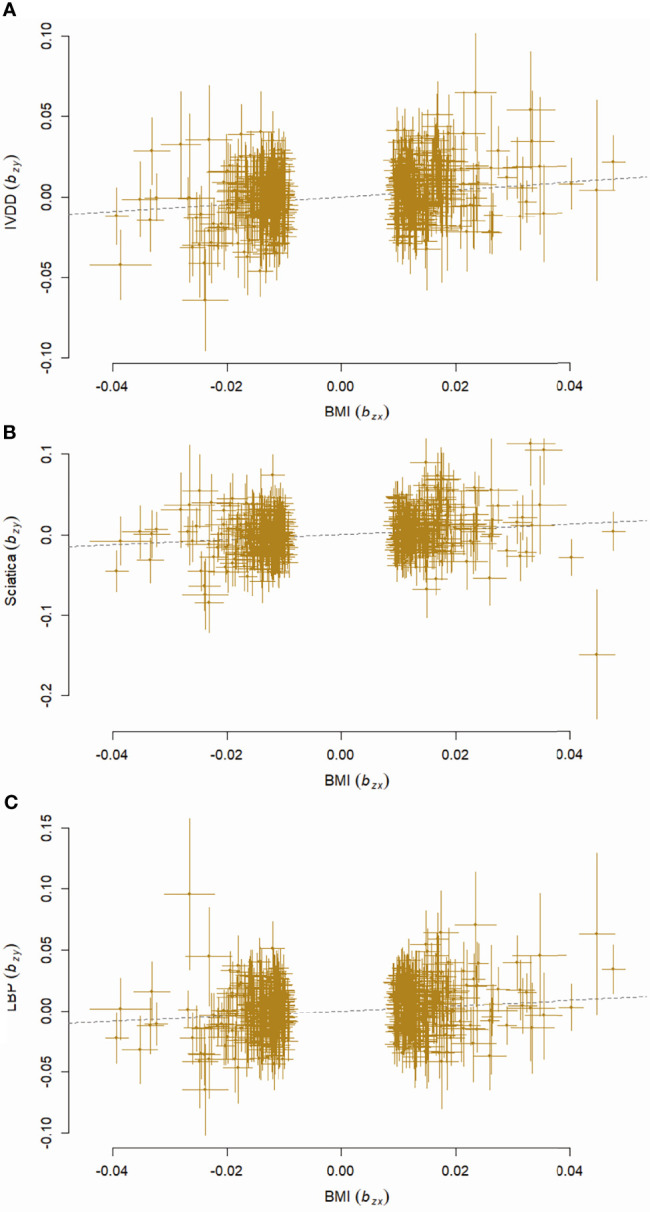
Scatter plots showing effects of BMI on IVDD **(A)**, sciatica **(B)**, and LBP **(C)** with GSMR analyses. BMI, body mass index; IVDD, intervertebral disc degeneration; LBP, low back pain.

The causal relationships between BMI and all the outcomes remained consistent with GSMR analyses. Genetically predicted BMI significantly increased the risk of IVDD, sciatica, and LBP with ORs equal to 1.25 (95% CI, 1.15–1.36, p = 2.75 × 10^-7^), 1.37 (95% CI, 1.22–1.55, p = 2.54 × 10^-7^), and 1.24 (95% CI, 1.12, 1.38, p = 2.81 × 10^-5^) (**Figure 5**).

## Discussion

In the current study, we investigated the causal relationships between BMI/obesity-related traits and different dorsopathies by using multiple MR methods. This study indicated that a higher BMI was an important risk factor causally associated with the various dorsopathies.

Previous studies agreed on whether elevated BMI, especially being overweight or obese, promotes the development of IVDD. Özcan-Ekşi et al. have shown that the severity of IVDD at L4-L5 and L5-S1 disc levels was closely associated with BMI in women with LBP. The women with severe IVDD at L4-L5 and L5-S1 disc levels were more overweight or obese when compared to those with mild-to-moderate IVDD, respectively (30415054) Recently, Özcan-Ekşi et al. evaluated 151 patients with LBP in terms of IVDD, end-plate changes, and fatty infiltration in the paraspinal muscles at all lumbar levels. They found that obese patients had more severe IVDD than non-obese patients at L4-L5. They suggested that the difference might be due to unbalanced shear forces at lower lumbar levels, particularly in women (37). A cohort study conducted by Liuke et al. found that the persistence of overweight (BMI ≥ 25 kg/m^2^) is strongly associated with the number of lumbar discs with decreased intensity of nucleus pulposus under magnetic resonance imaging (MRI) (18). Furthermore, they also noticed that overweight at a young age was positively correlated with the number of degenerated lumbar discs at middle age. These findings are consistent with our observations by using the MR method since the MR estimates the effects of a given exposure over a lifelong time (38).

A cross-sectional study conducted by Samartzis et al. has also shown that subjects in the group of disc degeneration have significantly higher BMI values (mean 23.3 kg/m^2^) compared to subjects without degeneration (mean 21.7 kg/m^2^) (19). Also, they showed that in the group with elevated BMI, there is a significant increase in the number of degenerated discs and the severity of degeneration. Similarly, a meta-analysis enrolling 1,749 patients with lumbar disc diseases and 1,885 controls suggested that overweight increased the risk of lumbar disease and is a predominant factor compared with age and sex with a subgroup analysis (39). Accumulating evidence has shown that endplate defect is an initiating factor that triggers IVDD (13–17). BMI has been suggested to contribute to IVDD through endplate defect (16).

On the contrary, several other studies provided evidence against such an association. Videman et al. performed an exposure-discordant twin study and reported that elevated BMI increased the bone mineral density of lumbar vertebrae, lumbar disc height, and intensity (21). A recent observational study enrolling 1,128 female twins found that BMI was weakly associated with chronic back pain in results from analyzing the total samples; however, the association disappeared after adjusting for the shared environment and genetic factors by using within-pair case–control analysis (40). Interestingly, they also assessed the effect of waist circumference and waist–hip ratio on chronic low back pain and noticed that higher waist-hip ratios appeared to be a protective factor for low back pain with an OR of 0.67 (95% CI 0.47–0.94) while no effect was found for waist circumference. The different observations may be due to the study design of the previous publications, which are mostly observational and cannot rule out the effect of confounders or reverse causality.

Several biomechanical studies quantified the mechanical forces exerted on low back and intervertebral disc. Singh et al. demonstrated that in severely obese patients (BMI ≥ 35 kg/m2), the L5/S1 disc compression forces exceeded the 3,400-N NIOSH action limit (range from 3,000 to 8,500 N) (41). Meanwhile, the group mean disc compression force is significantly higher in severely obese patients as compared with the normal-weight group (41). Coppock et al. developed non-invasive magnetic resonance imaging and solid modeling techniques to measure the *in vivo* intervertebral disc (IVD) deformation following treadmill walking stress test, and the study showed the highest statistical significant association between BMI and compressive deformation in the L5-S1 IVD (R2 = 0.61, p < 0.05), and the association became weaker in the L3-L4 and L4-L5 IVDs (42). Another study showed that as body weight increased from 51 to 119 kg, the L5-S1 compression increased by ~80%–147% with no load in hands and by ~46%–52% in load holding tasks in flexed postures. In addition, spinal loads further increased by up to 15% in severely obese individuals (BMI > 30 kg/m2) (43). In summary, obese patients are at a higher risk of IVDD-associated outcomes due to higher mechanical forces exerted on lumbar IVD in both normal and mechanical loading conditions.

In this current study, we assessed the causal effect of waist circumference, hip circumference, and waist–hip ratio on IVDD, sciatica, and LBP with MR methods. A significant effect on IVDD was observed with waist circumference and hip circumference, but not with waist–hip ratio. Similarly, higher waist and hip circumference increased the risk of sciatica with lumbago in our analysis. The causal effect of waist and hip circumference on IVDD disappeared after the adjustment for BMI with multivariable MR, while whole body fat-free mass remained consistent. This might be due to the high correlation between body fat-free mass/muscle mass and heavy physical labor which is another risk factor for IVDD.

Damaged lumbar intervertebral disc and endplates can lead to compression on nerve roots, resulting in traumatic neuropathic pain (9). Shiri et al. found that overweight and obesity were positively correlated with the risk of hospitalization for sciatica and lumbar radicular pain in a meta-analysis of 26 studies (44). Similarly, a recent meta-analysis of 10 cohort studies including 29,748 subjects has indicated that overweight and obesity are risk factors for the development of LBP in both genders (45). These findings are consistent with our results from MR analyses that genetically proxied BMI is positively correlated with the risk of sciatica and LBP. Even though other body composition factors also showed causal relationships with sciatica and LBP, these relationships disappeared after adjusting for the effect of BMI (**Figures 2**–**4**).

Since BMI is highly related to sedentary behaviors, we have also performed a mediation analysis to estimate the proportion of BMI’s effect mediated by leisure sedentary behavior (television watching). We showed that 41.4% (95% CI, 21.8%, 64.8%), 33.8% (95% CI, 17.5%, 53.4%), and 49.7% (95% CI, 29.4%, 73.5%) of BMI’s effect on IVDD, sciatica, and LBP were indirect effects mediated by sedentary behavior. These results suggested that BMI posed a deleterious effect on IVDD, sciatica, and LBP independent of leisure sedentary behavior.

The first advantage of using the MR method is that we could study the effect of BMI on IVDD, sciatica, and LBP by using publicly available large GWAS datasets. Since genetic variant alleles are assigned randomly, the causal estimates are less prone to confounding factors and reverse causality. Furthermore, bias because of population stratification could be mitigated by confining the study population to European ancestry.

However, there are several limitations to this study. Firstly, distortion of estimates from MR analyses may happen due to the existence of pleiotropy, which is when one genetic instrument is associated with more phenotypes other than the exposure of interest. We have used MR-Egger as sensitivity analysis for detecting potential pleiotropy, and no significant pleiotropy was identified.

In conclusion, our comprehensive MR study provides evidence that higher BMI is causally associated with the risk of IVDD, sciatica, and LBP. Weight control should be considered in populations with obesity to reduce the risk of dorsopathies including IVDD, sciatica, and LBP.

## Data Availability Statement

Publicly available datasets were analyzed in this study. These data can be found here as follows: the sources of GWAS data included in the study are presented in the article/**Supplementary Tables 1**, **2**. Further inquiries can be directed to the corresponding authors.

## Ethics Statement

Ethical review and approval were not required for the study on human participants in accordance with the local legislation and institutional requirements. Written informed consent for participation was not required for this study in accordance with the national legislation and the institutional requirements.

## Author Contributions

ZL and HH conceptualized and designed the study. ZL, JZ, JM, and YP performed data analysis. ZL, JZ, and JM wrote the manuscript. All authors contributed to the article and approved the submitted version.

## Funding

This study was funded by the Nature Science Foundation of Shandong Province (No. ZR2016HL11) and Undergraduate Training Program for Innovation and Entrepreneurship of Shandong Province (S202010438060).

## Conflict of Interest

The authors declare that the research was conducted in the absence of any commercial or financial relationships that could be construed as a potential conflict of interest.

## Publisher’s Note

All claims expressed in this article are solely those of the authors and do not necessarily represent those of their affiliated organizations, or those of the publisher, the editors and the reviewers. Any product that may be evaluated in this article, or claim that may be made by its manufacturer, is not guaranteed or endorsed by the publisher.

## Supplementary Material

Supplementary Figure 1 Diagram of the study design with the three assumptions of Mendelian Randomization study.

Supplementary Table 1 Data source and detailed information of the included exposures.

Supplementary Table 2 Data source and detailed information of the included outcomes.

Supplementary Table 3 Results from sensitivity analyses.
