# Supplementary figures and images for: Causal Associations of Obesity With the Intervertebral Degeneration, Low Back Pain, and Sciatica: A Two-Sample Mendelian Randomization Study
Source: Front Endocrinol (Lausanne). 2021 Dec 8;12:740200. doi: 10.3389/fendo.2021.740200 (PMC8692291; doi:10.3389/fendo.2021.740200)

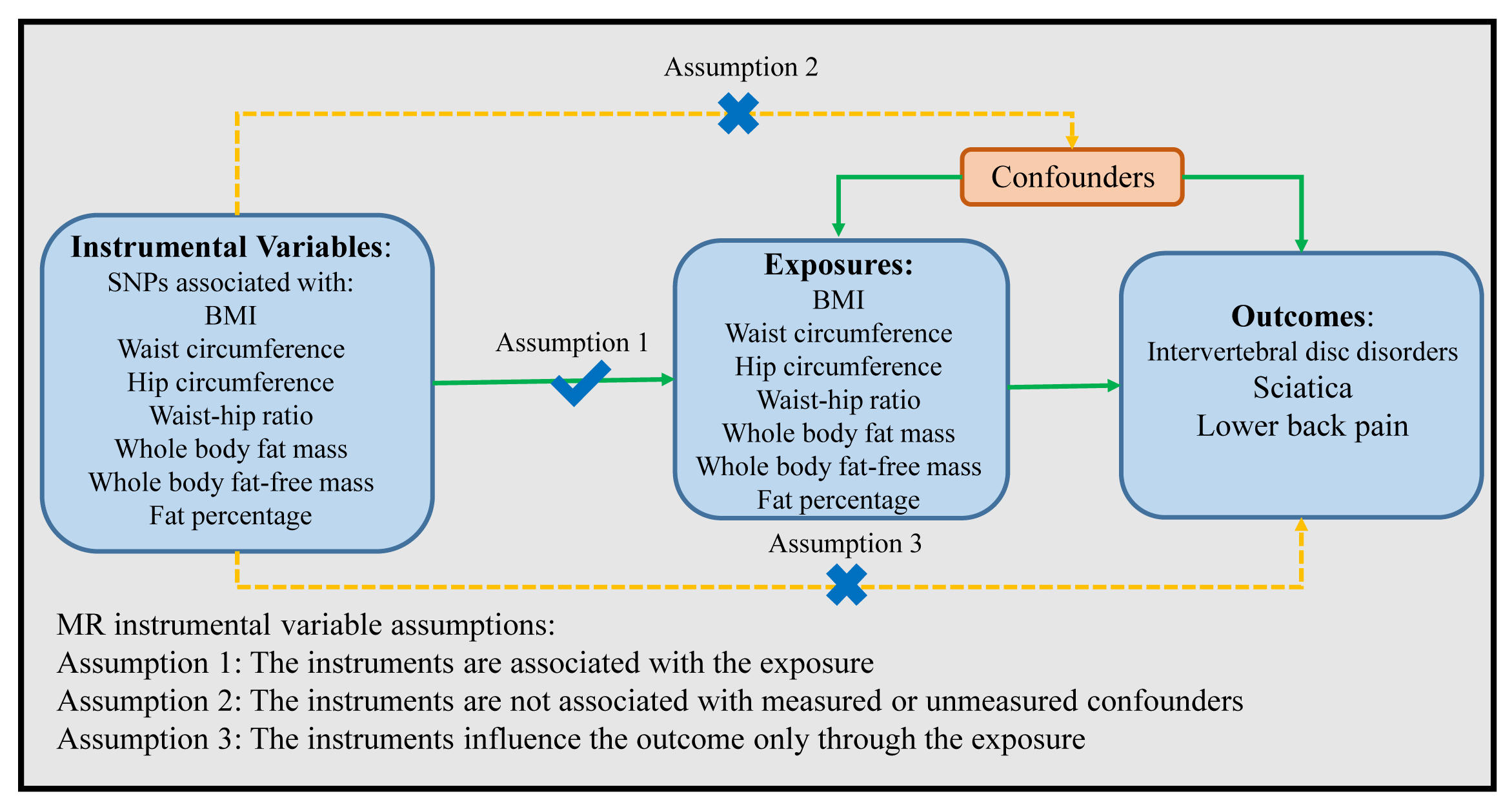

Supplement: Supplementary Figure 1 — Diagram of the study design with the three assumptions of Mendelian Randomization study. [file Image_1.tif]
