# Supplementary material for: Causal Associations of Obesity With the Intervertebral Degeneration, Low Back Pain, and Sciatica: A Two-Sample Mendelian Randomization Study
Source: Front Endocrinol (Lausanne). 2021 Dec 8;12:740200. doi: 10.3389/fendo.2021.740200 (PMC8692291; doi:10.3389/fendo.2021.740200)

Supplementary Figure 2: Scatter plot of the causal effect of BMI on IVDD.


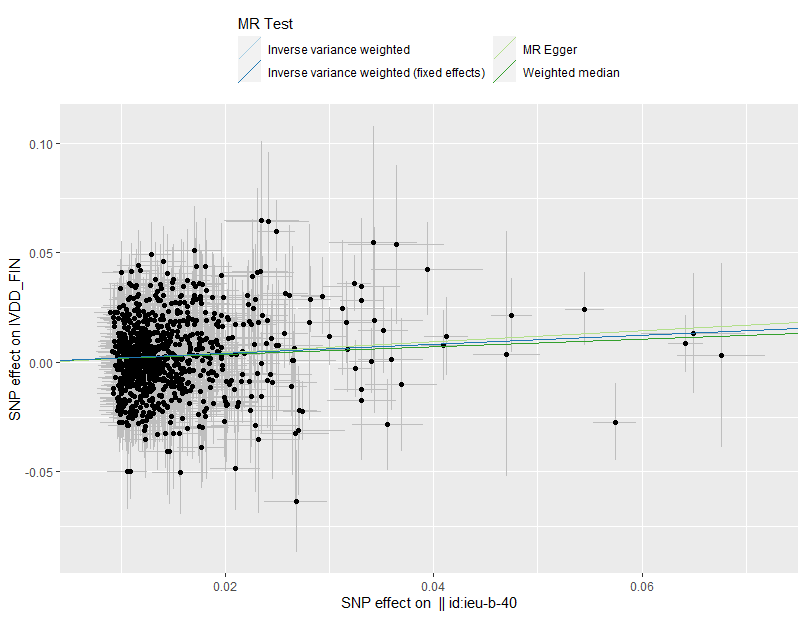


Supplementary Figure 3: Scatter plot of the causal effect of waist circumference on IVDD.


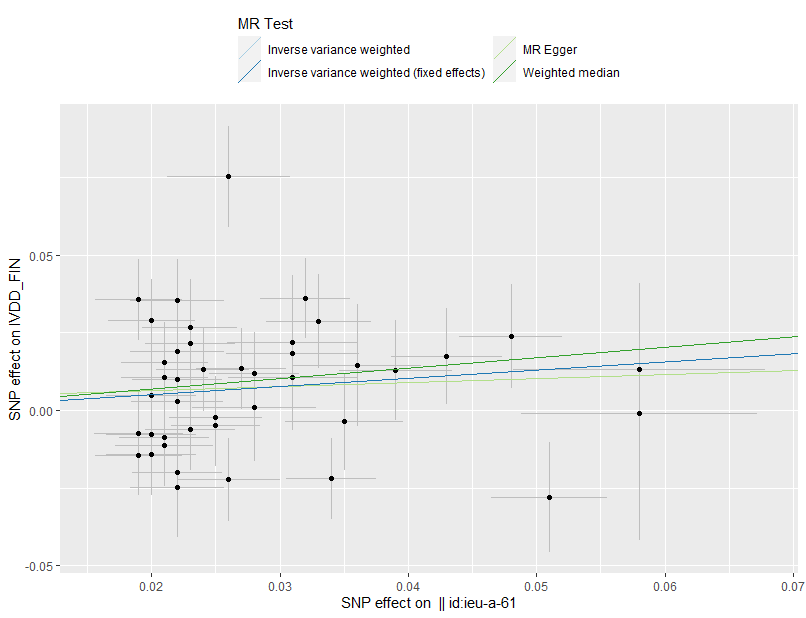


Supplementary Figure 4: Scatter plot of the causal effect of hip circumference on IVDD.


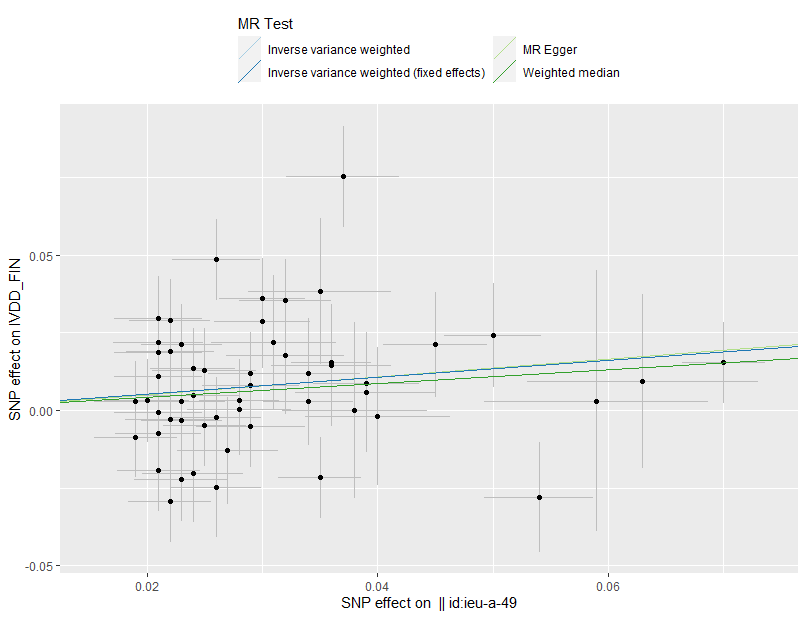


Supplementary Figure 5: Scatter plot of the causal effect of waist-hip ratio on IVDD.


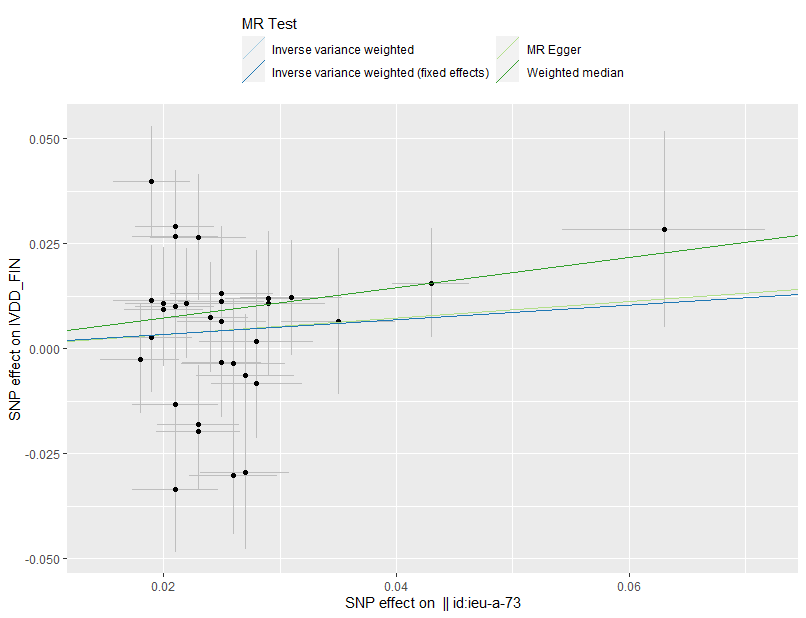


Supplementary Figure 6: Scatter plot of the causal effect of whole-body fat mass on IVDD.
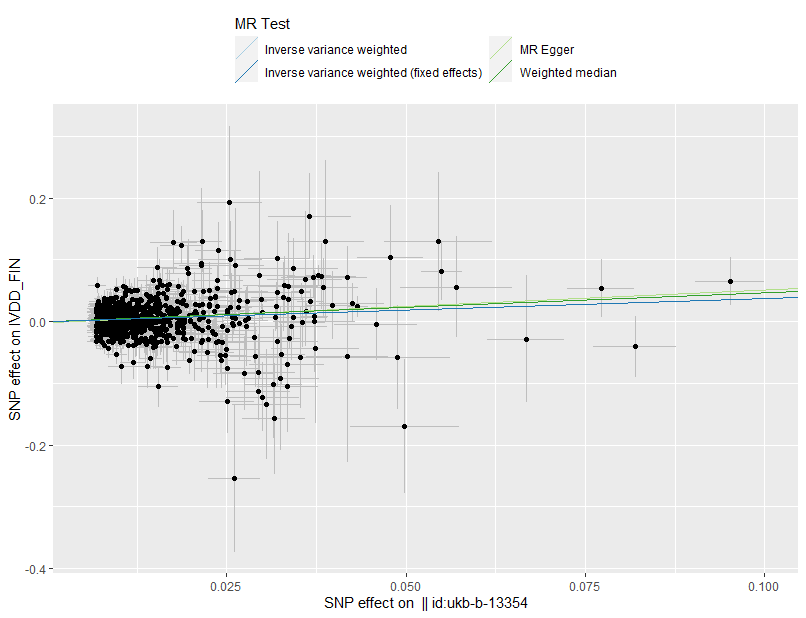


Supplementary Figure 7: Scatter plot of the causal effect of whole-body fat-free mass on IVDD.


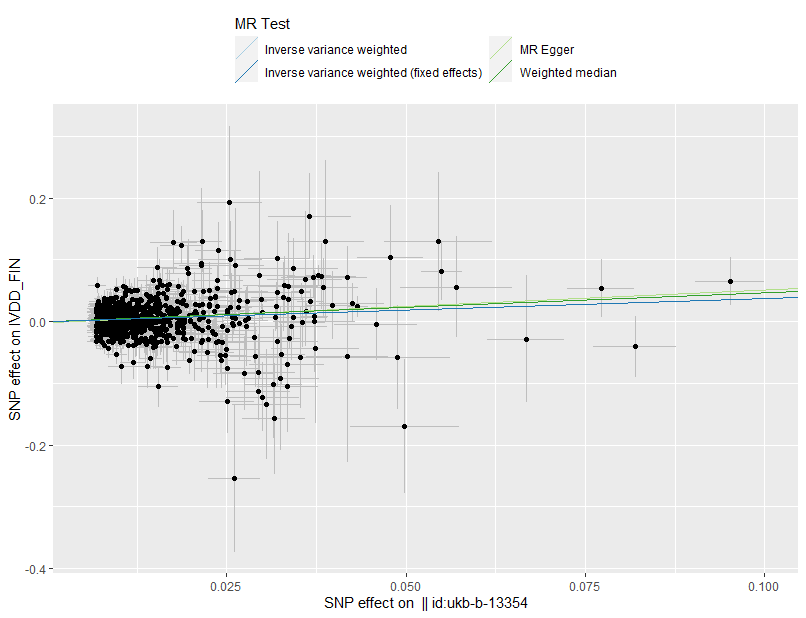


Supplementary Figure 8: Scatter plot of the causal effect of whole-body fat percentage on IVDD.


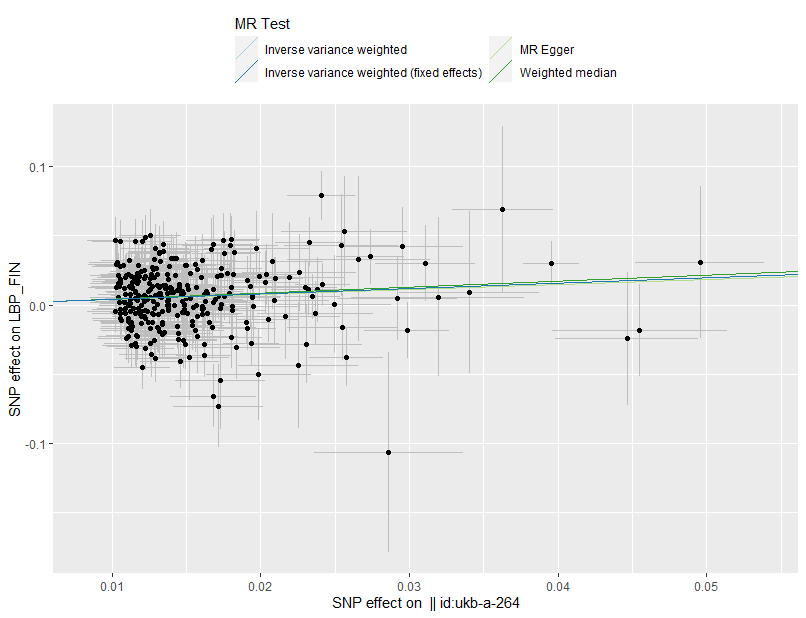


Supplementary Figure 9: Scatter plot of the causal effect of BMI on sciatica.


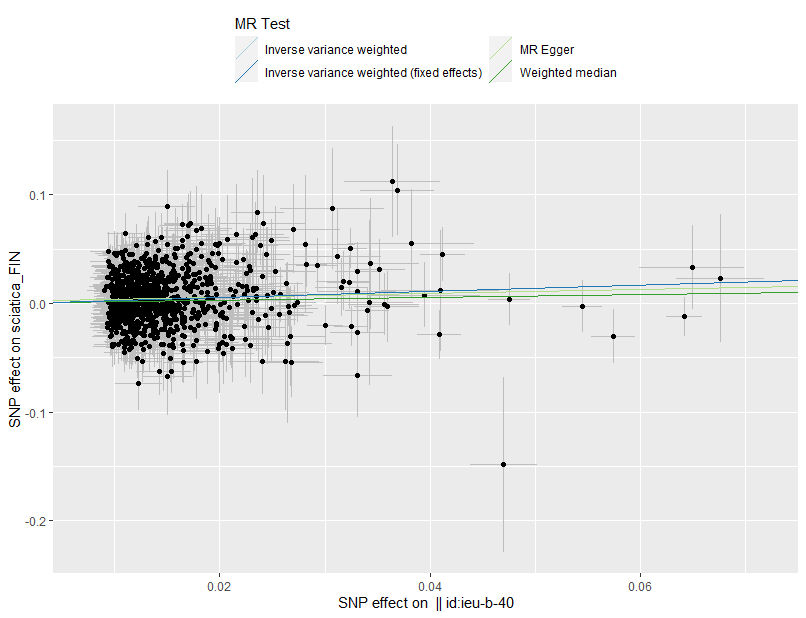


Supplementary Figure 10: Scatter plot of the causal effect of waist circumference on sciatica.


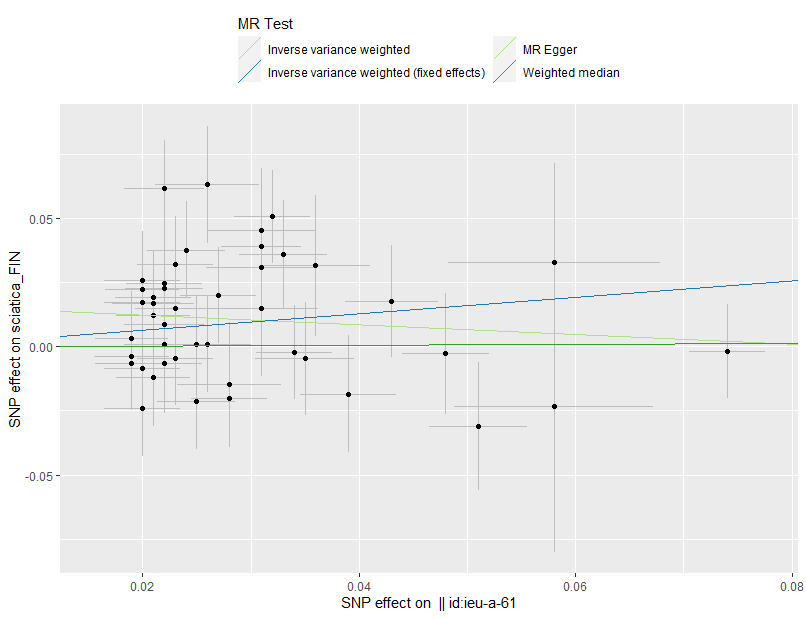


Supplementary Figure 1: Scatter plot of the causal effect of hip circumference on sciatica.


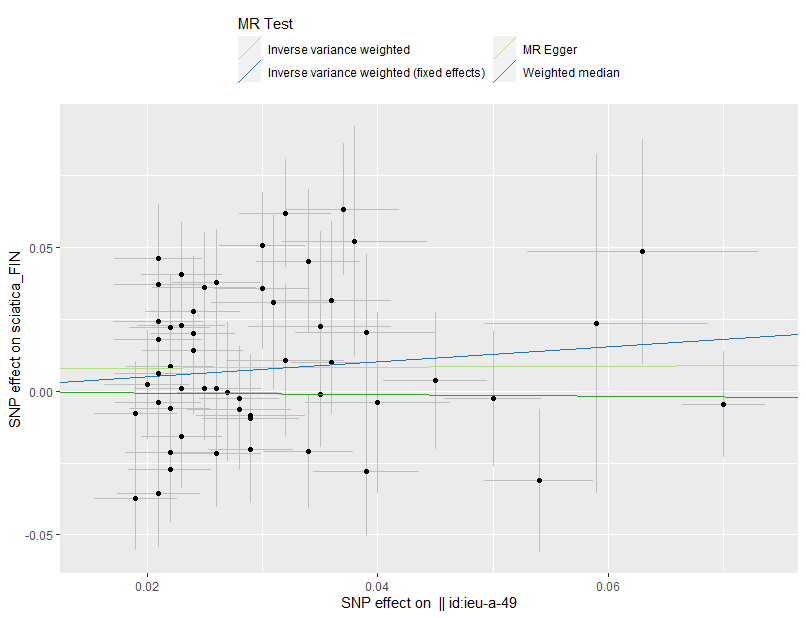


Supplementary Figure 12: Scatter plot of the causal effect of waist-hip ratio on sciatica.


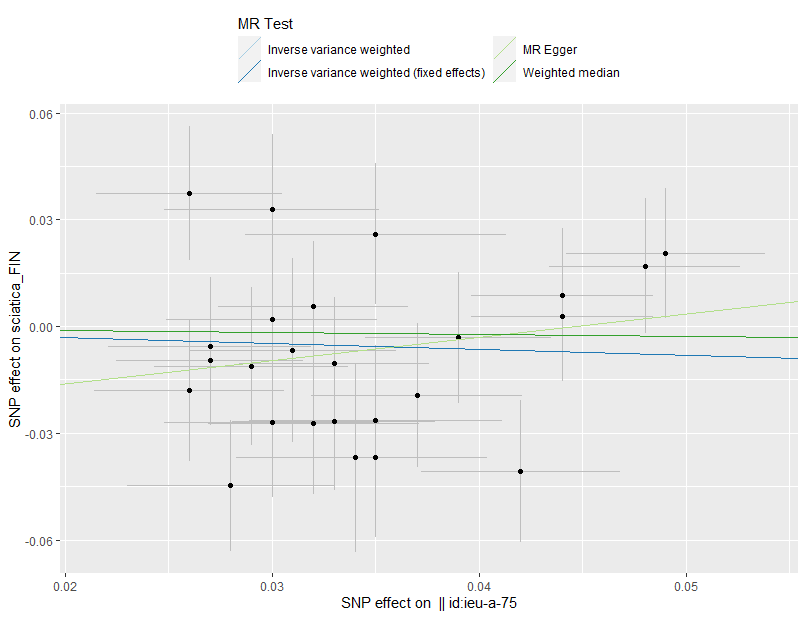


Supplementary Figure 13: Scatter plot of the causal effect of whole-body fat mass on sciatica.


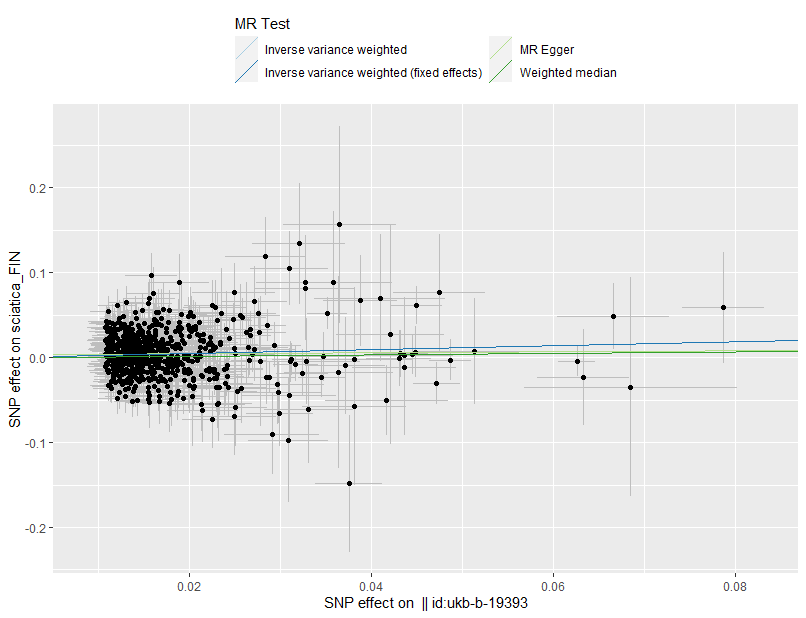


Supplementary Figure 14: Scatter plot of the causal effect of whole-body fat-free mass on sciatica.


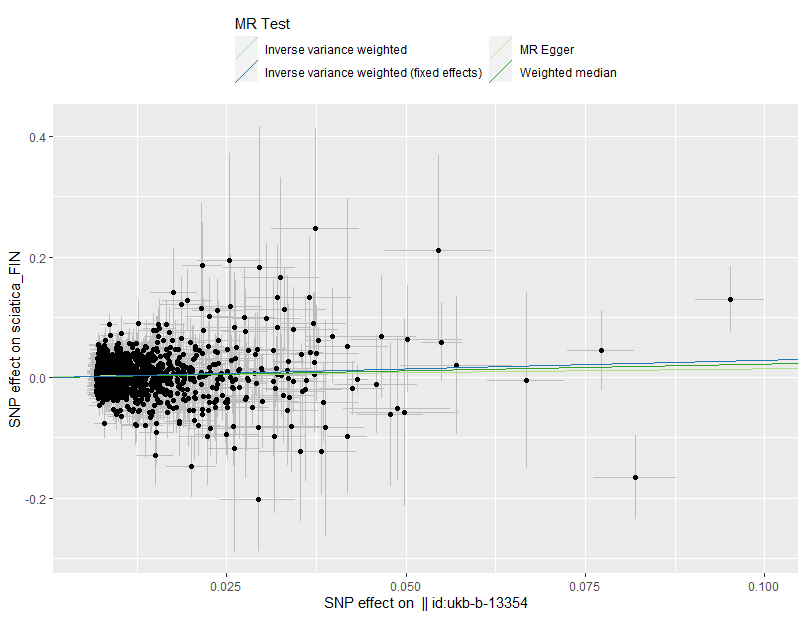


Supplementary Figure 15: Scatter plot of the causal effect of whole-body fat percentage on sciatica.


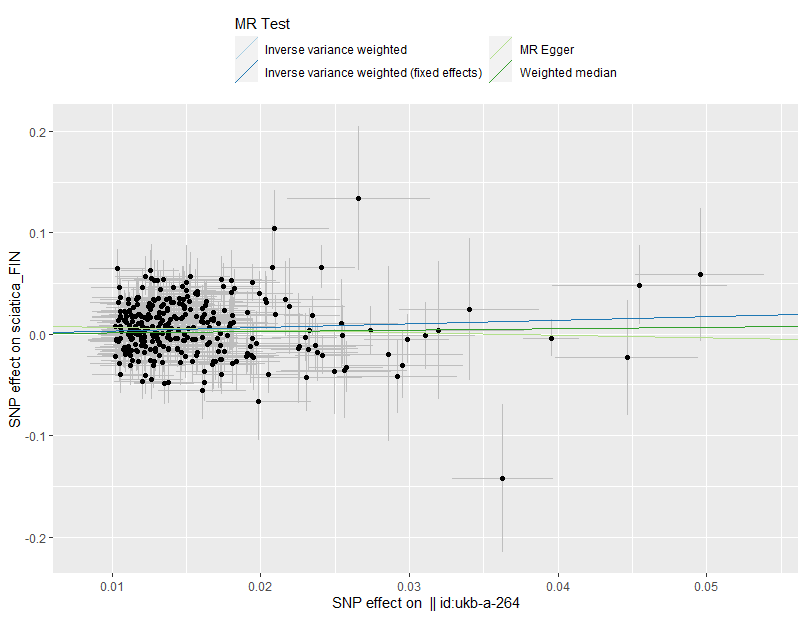


Supplementary Figure 16: Scatter plot of the causal effect of BMI on LBP.


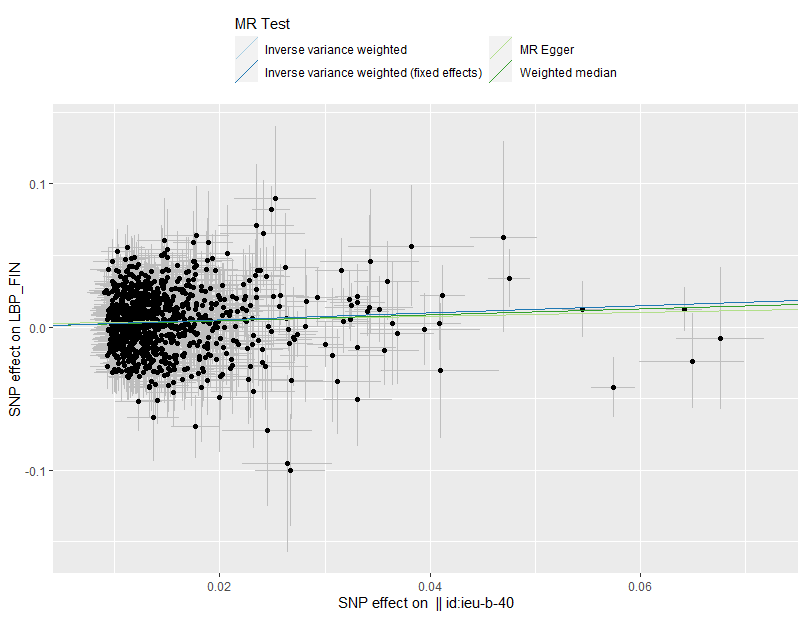


Supplementary Figure 17: Scatter plot of the causal effect of waist circumference on LBP.


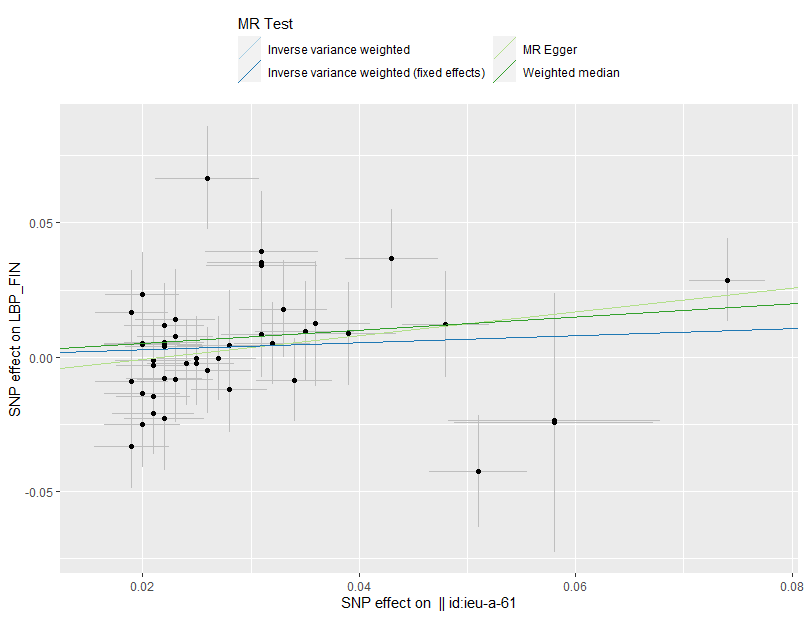


Supplementary Figure 18: Scatter plot of the causal effect of hip circumference on LBP.


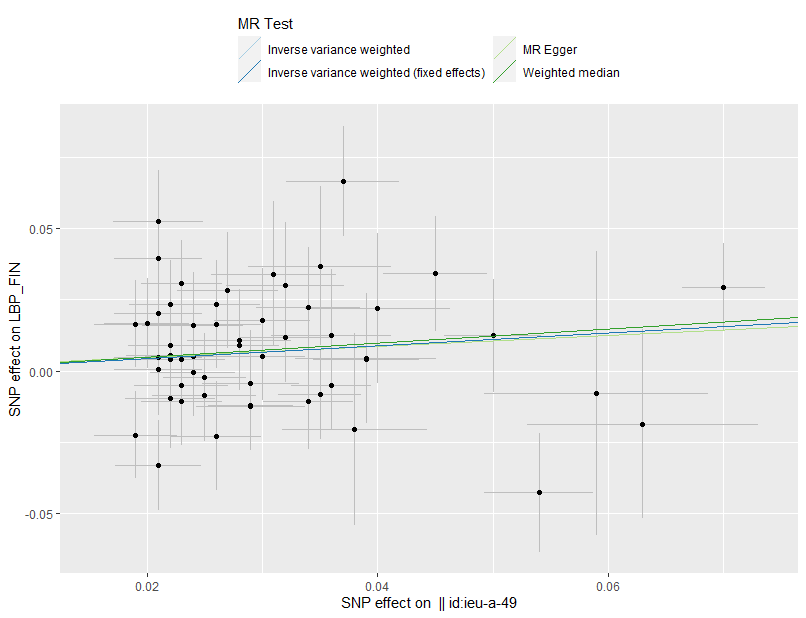


Supplementary Figure 19: Scatter plot of the causal effect of waist-hip ratio on LBP.


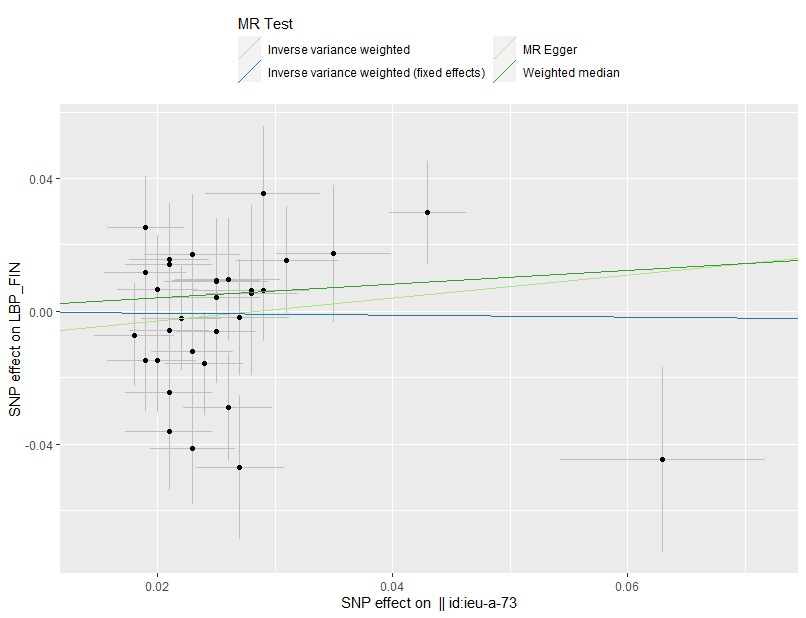


Supplementary Figure 20: Scatter plot of the causal effect of whole-body fat mass on LBP.


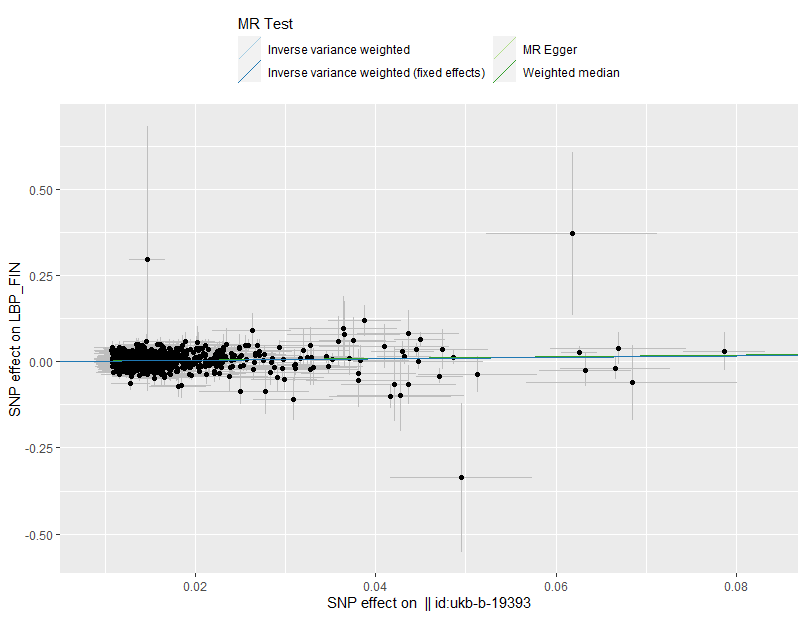


Supplementary Figure 21: Scatter plot of the causal effect of whole-body fat-free mass on LBP.


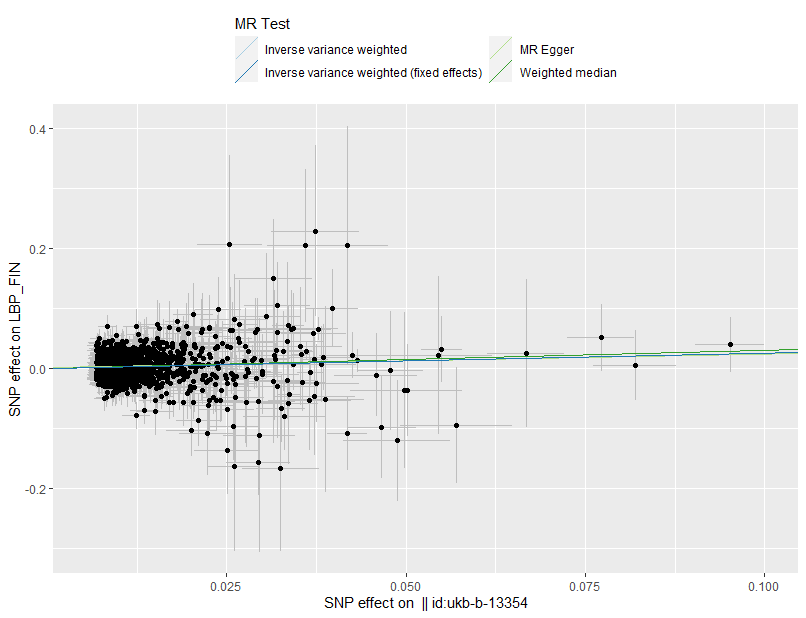


Supplementary Figure 22: Scatter plot of the causal effect of whole-body fat percentage on LBP.


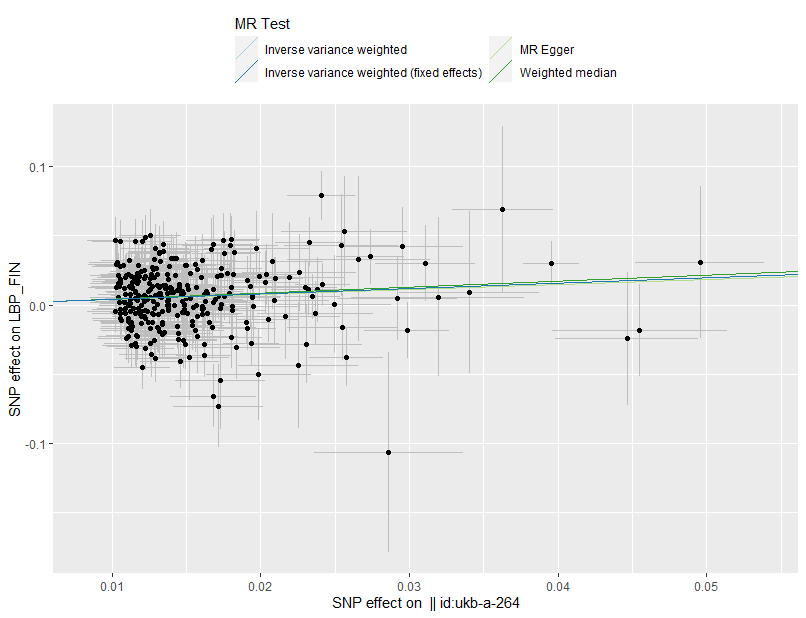

Supplement: Supplementary file 5 [file DataSheet_1.docx]
