# Supplementary material for: Causal Associations of Obesity With the Intervertebral Degeneration, Low Back Pain, and Sciatica: A Two-Sample Mendelian Randomization Study
Source: Front Endocrinol (Lausanne). 2021 Dec 8;12:740200. doi: 10.3389/fendo.2021.740200 (PMC8692291; doi:10.3389/fendo.2021.740200)

Supplementary Figure 23: Funnel plot of the causal effect of BMI on IVDD.


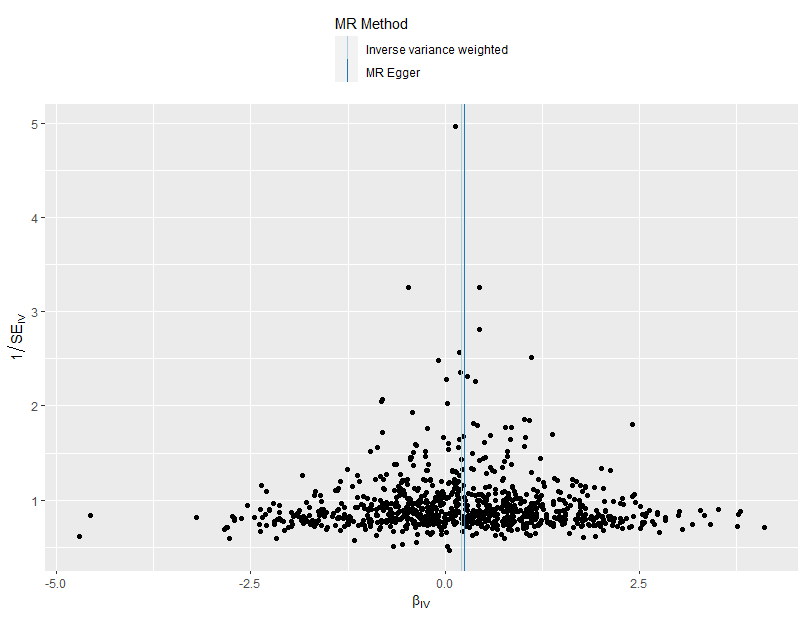


Supplementary Figure 24: Funnel plot of the causal effect of waist circumference on IVDD.


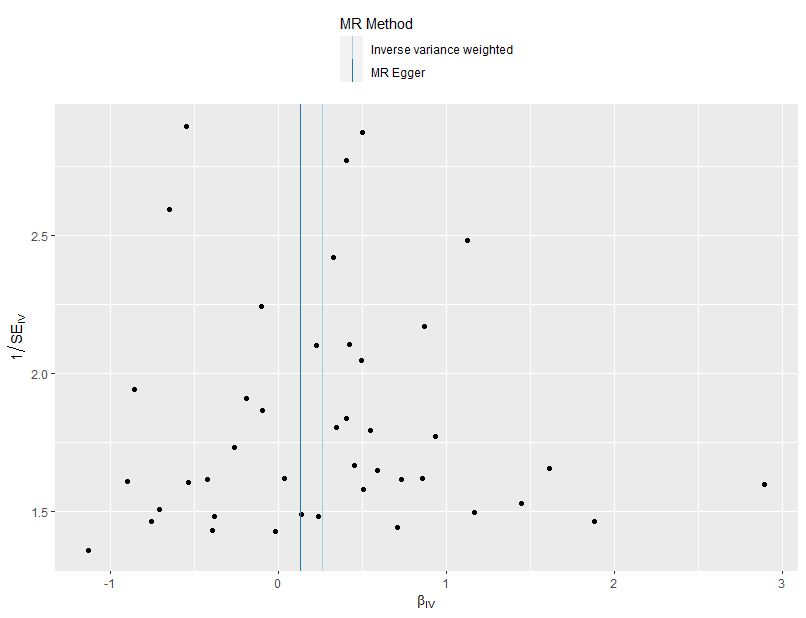


Supplementary Figure 25: Funnel plot of the causal effect of hip circumference on IVDD.


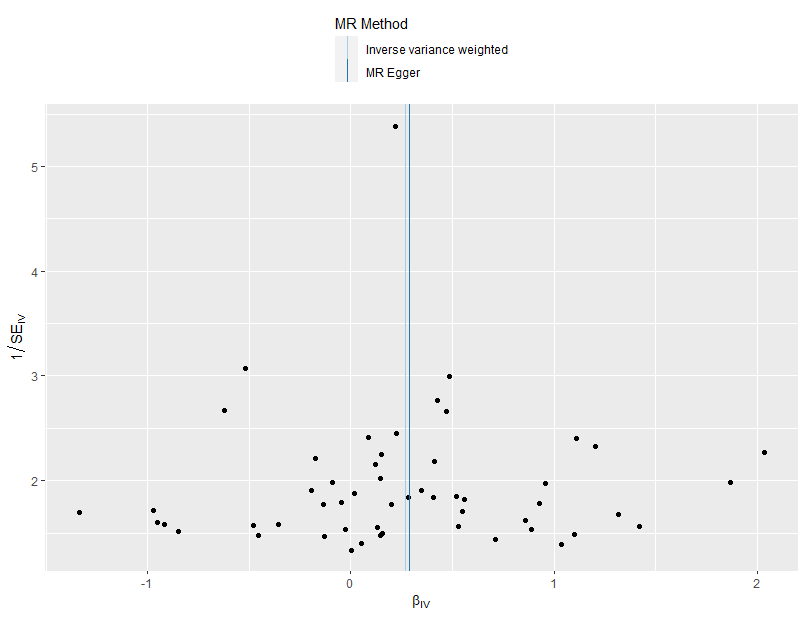


Supplementary Figure 26: Funnel plot of the causal effect of waist-hip ratio on IVDD.


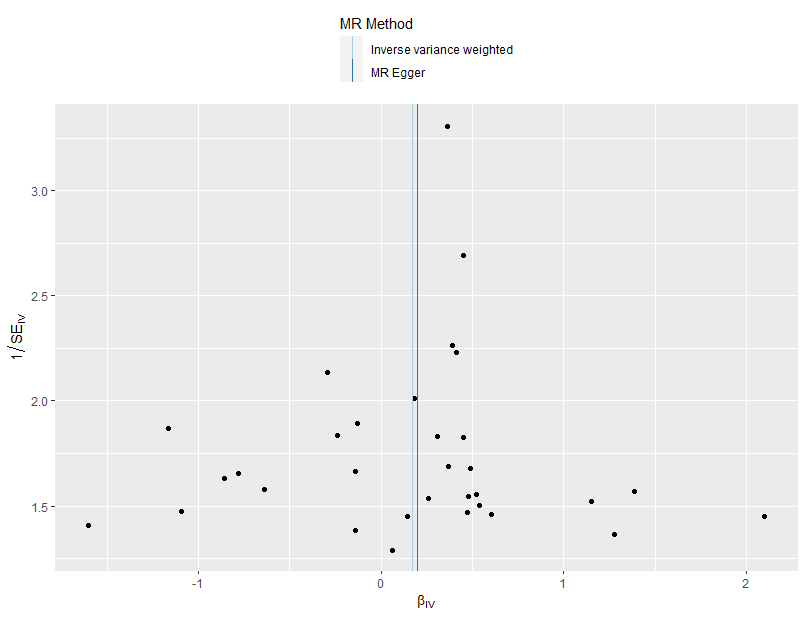


Supplementary Figure 27: Funnel plot of the causal effect of whole-body fat mass on IVDD.


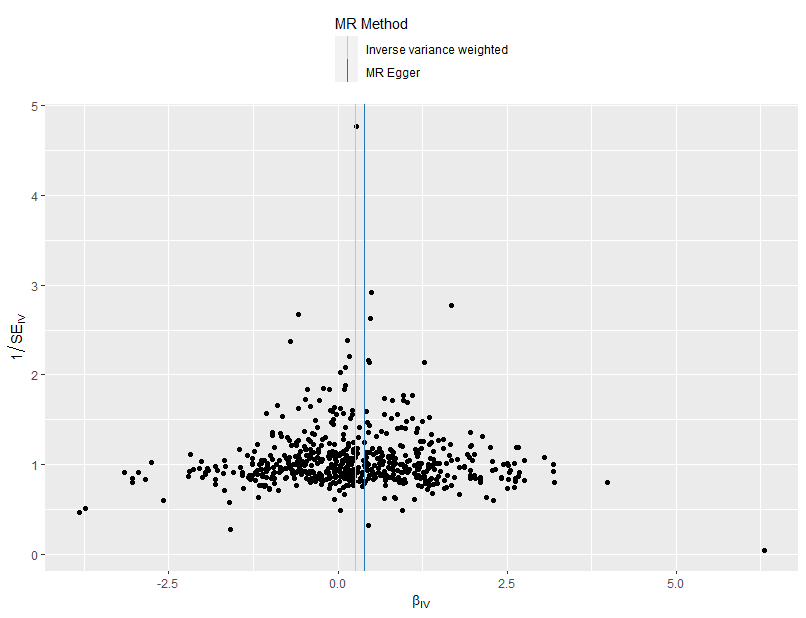


Supplementary Figure 28: Funnel plot of the causal effect of whole-body fat-free mass on IVDD.


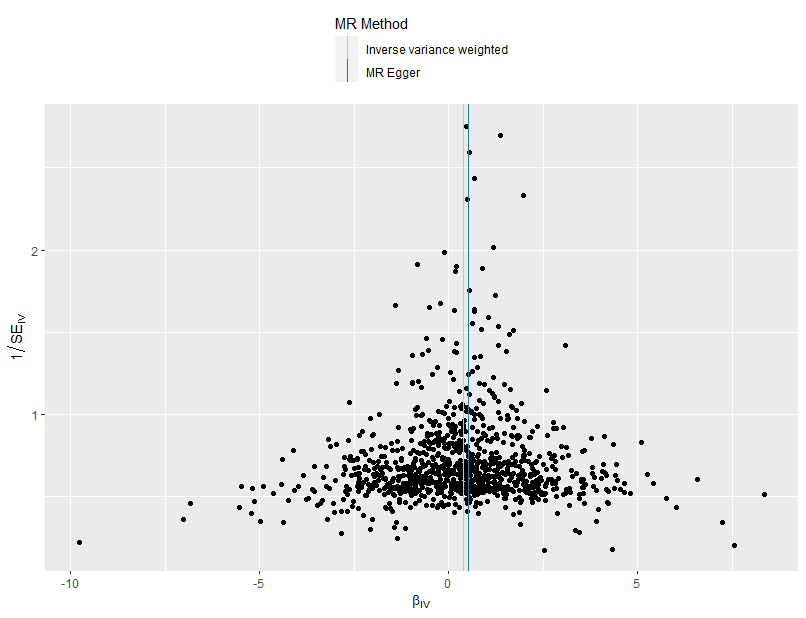


Supplementary Figure 29: Funnel plot of the causal effect of whole-body fat percentage on IVDD.


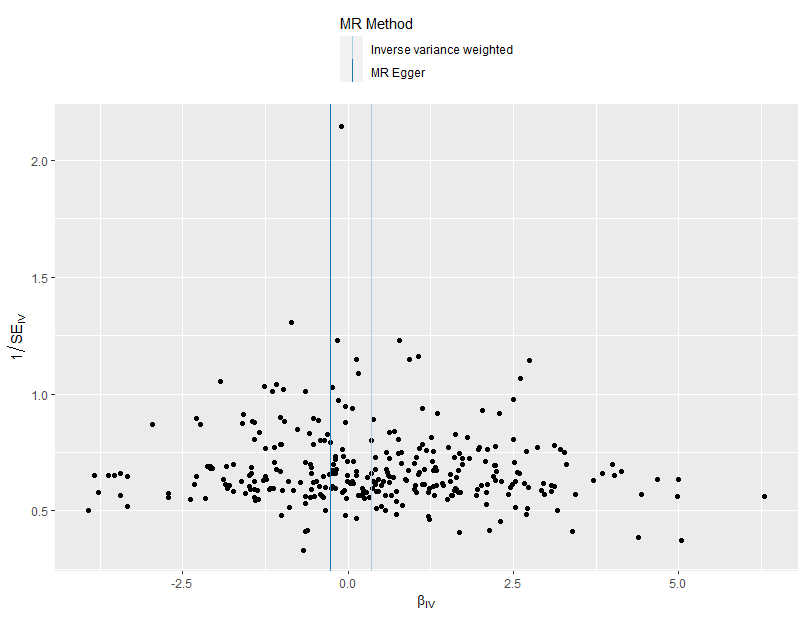


Supplementary Figure 30: Funnel plot of the causal effect of BMI on sciatica.


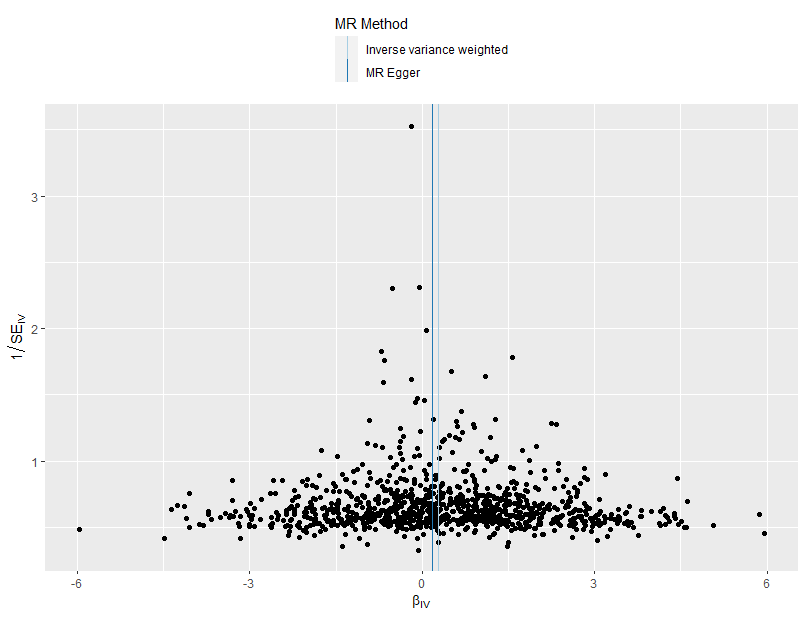


Supplementary Figure 31: Funnel plot of the causal effect of waist circumference on sciatica.


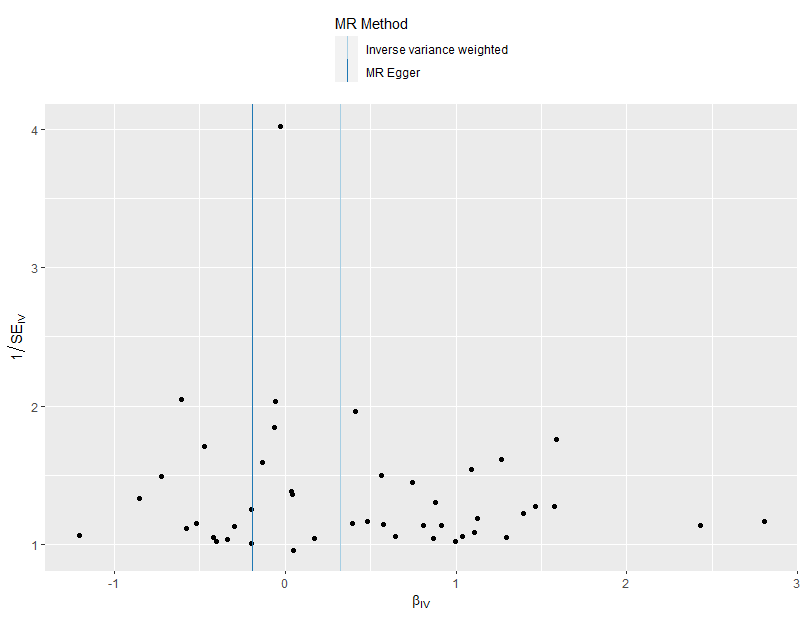
Supplementary Figure 32: Funnel plot of the causal effect of hip circumference on sciatica.


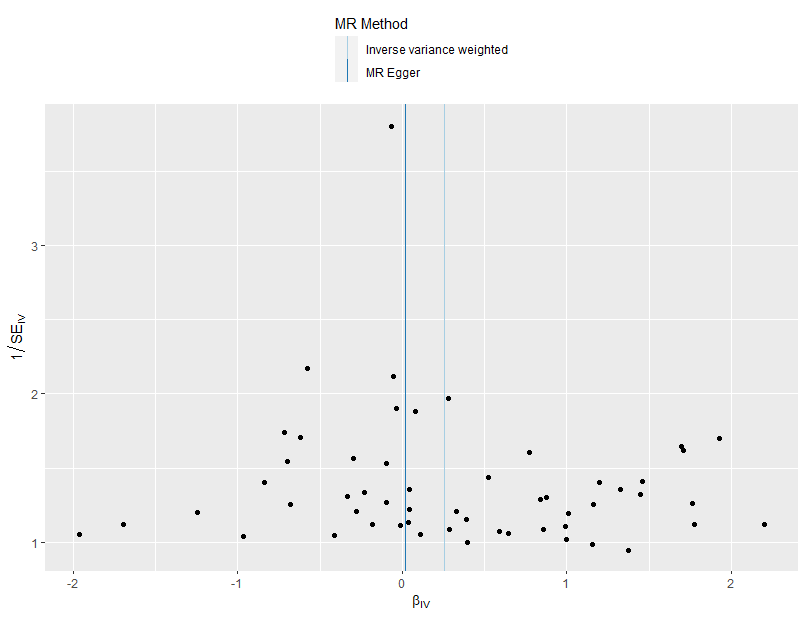


Supplementary Figure 33: Funnel plot of the causal effect of waist-hip ratio on sciatica.


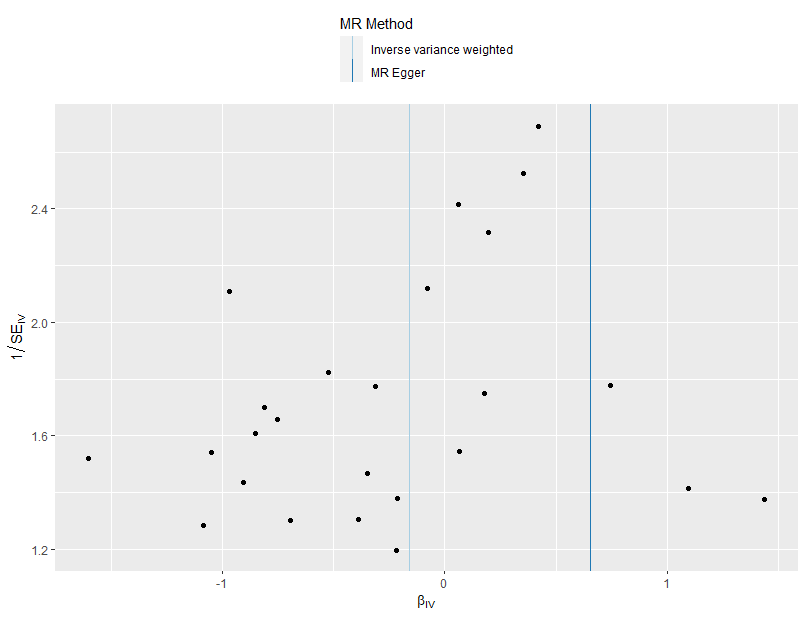


Supplementary Figure 34: Funnel plot of the causal effect of whole-body fat mass on sciatica.


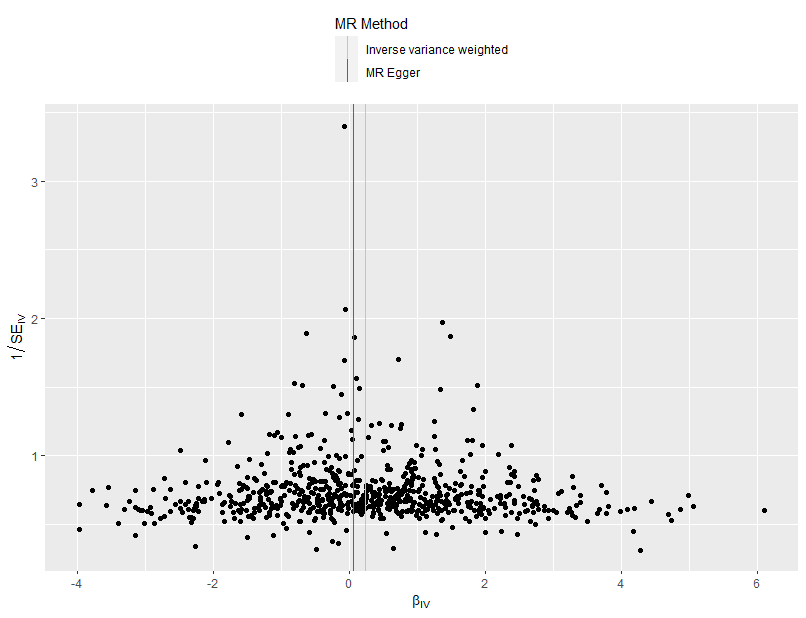


Supplementary Figure 35: Funnel plot of the causal effect of whole-body fat-free mass on sciatica.


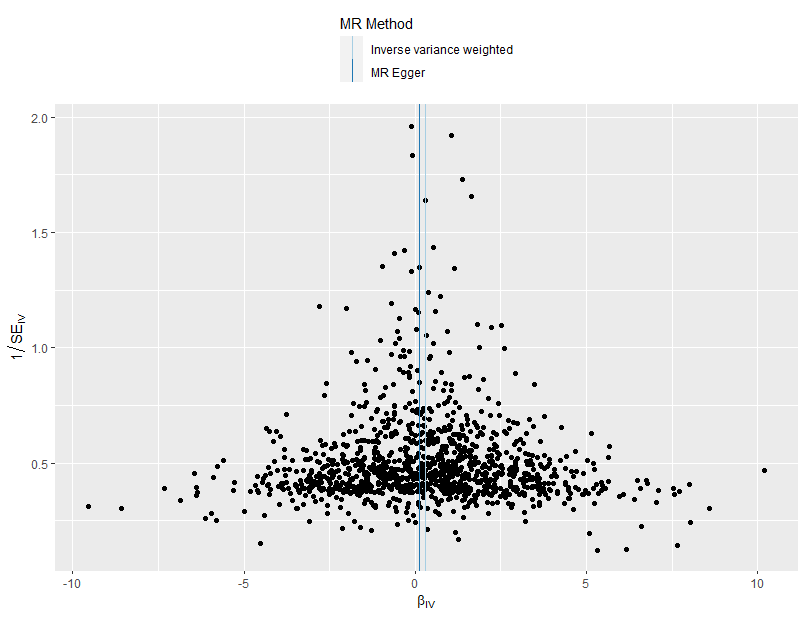


Supplementary Figure 36: Funnel plot of the causal effect of whole-body fat percentage on sciatica.


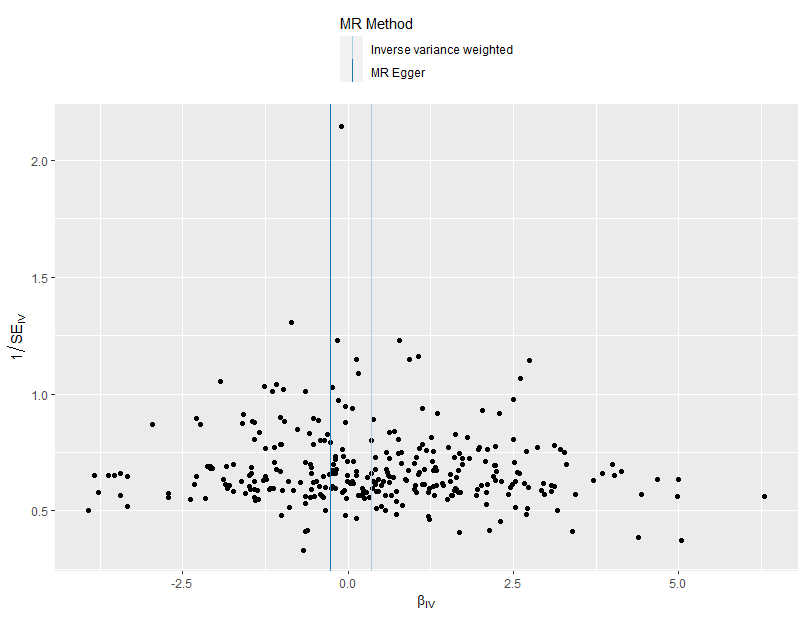


Supplementary Figure 37: Funnel plot of the causal effect of BMI on LBP.


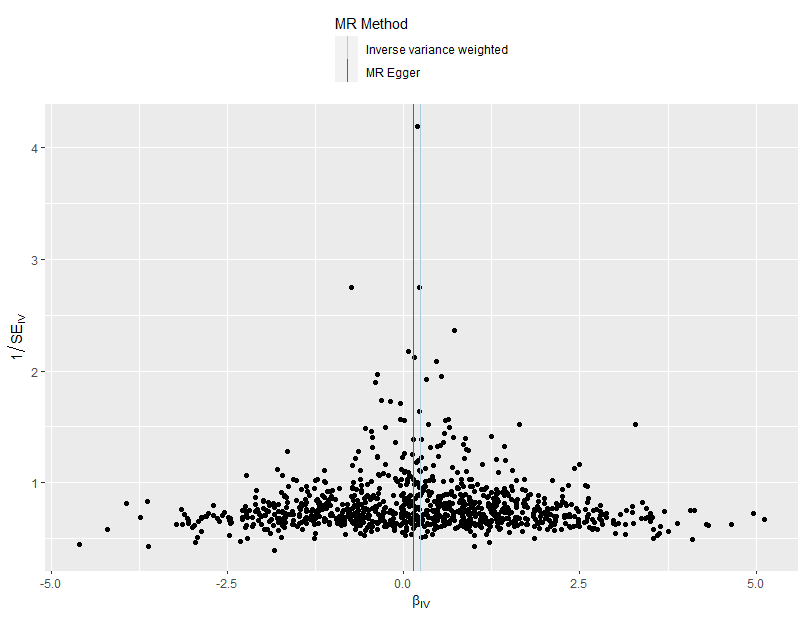


Supplementary Figure 38: Funnel plot of the causal effect of waist circumference on LBP.


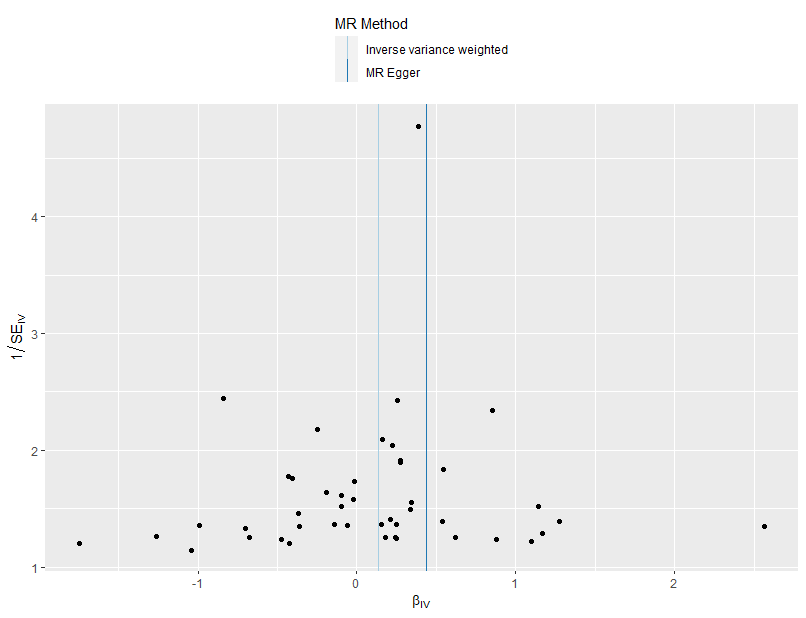


Supplementary Figure 39: Funnel plot of the causal effect of hip circumference on LBP.


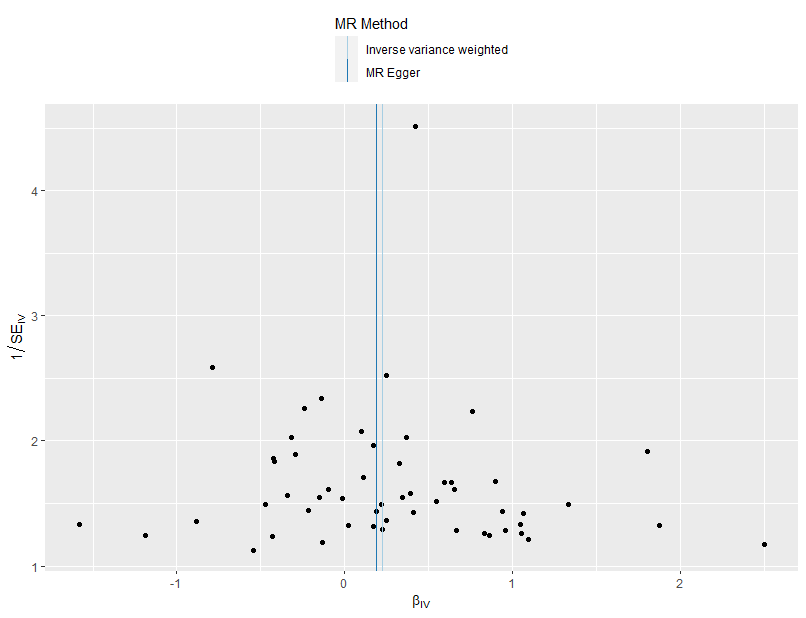


Supplementary Figure 40: Funnel plot of the causal effect of waist-hip ratio on LBP.


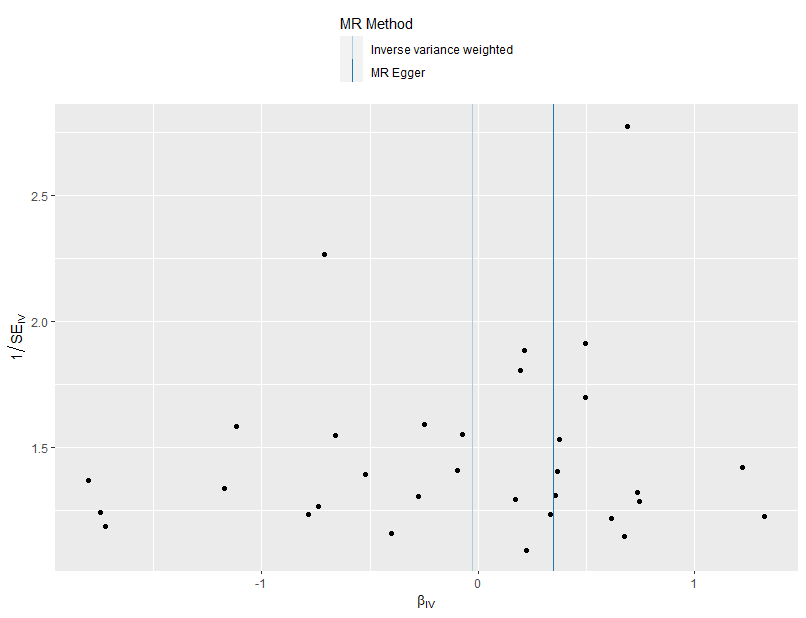


Supplementary Figure 41: Funnel plot of the causal effect of whole-body fat mass on LBP.


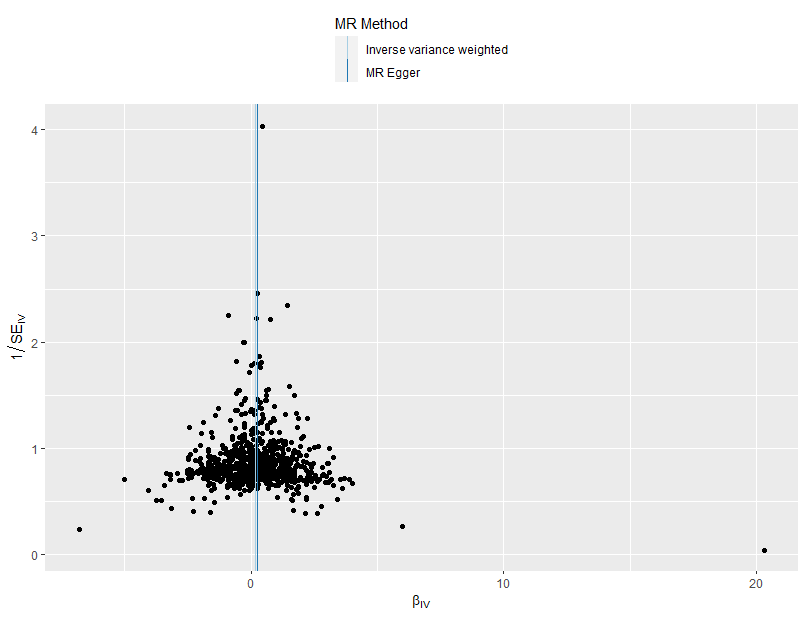


Supplementary Figure 42: Funnel plot of the causal effect of whole-body fat-free mass on LBP.


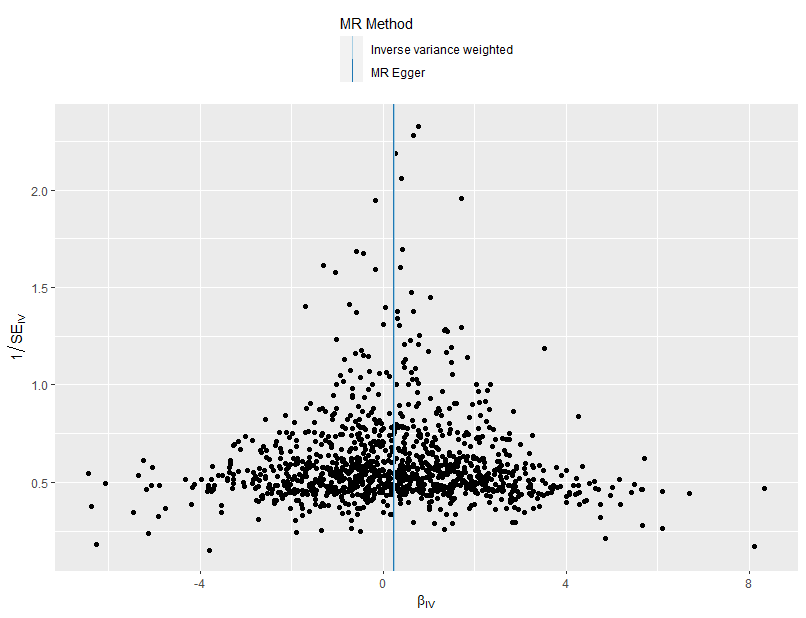


Supplementary Figure 43: Funnel plot of the causal effect of whole-body fat percentage on LBP.


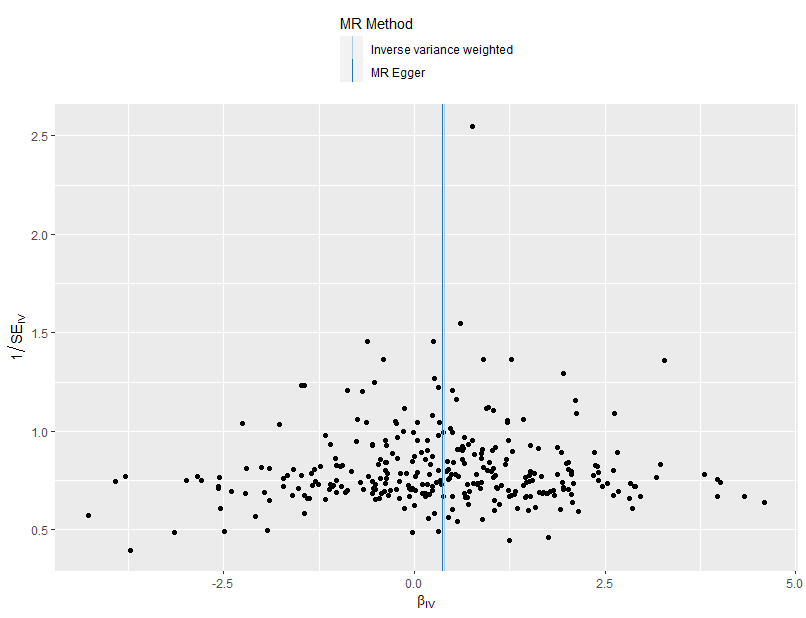

Supplement: Supplementary file 6 [file DataSheet_2.docx]
